# *Burkholderia multivorans* requires species‐specific GltJK for entry of a contact‐dependent growth inhibition system protein

**DOI:** 10.1111/mmi.14783

**Published:** 2021-07-28

**Authors:** Tanya Myers‐Morales, Martha M. S. Sim, Tanner J. DuCote, Erin C. Garcia

**Affiliations:** ^1^ Department of Microbiology, Immunology, and Molecular Genetics University of Kentucky Lexington KY USA; ^2^ Present address: Department of Molecular and Cellular Biochemistry University of Kentucky Lexington KY USA; ^3^ Present address: Department of Toxicology and Cancer Biology University of Kentucky Lexington KY USA

**Keywords:** bacterial antagonism, bacterial competition, CDI

## Abstract

Interbacterial antagonism and communication are driving forces behind microbial community development. In many Gram‐negative bacteria, contact‐dependent growth inhibition (CDI) systems contribute to these microbial interactions. CDI systems deliver the toxic C‐terminus of a large surface exposed protein to the cytoplasm of neighboring bacteria upon cell−contact. Termed the BcpA‐CT, import of this toxic effector domain is mediated by specific, yet largely unknown receptors on the recipient cell outer and inner membranes. In this study, we demonstrated that cytoplasmic membrane proteins GltJK, components of a predicted ABC‐type transporter, are required for entry of CDI system protein BcpA‐2 into *Burkholderia multivorans* recipient cells. Consistent with current CDI models, *gltJK* were also required for recipient cell susceptibility to a distinct BcpA‐CT that shared sequences within the predicted “translocation domain” of BcpA‐2. Strikingly, this translocation domain showed low sequence identity to the analogous region of an *Escherichia coli* GltJK‐utilizing CDI system protein. Our results demonstrated that recipient bacteria expressing *E. coli gltJK* were resistant to BcpA‐2‐mediated interbacterial antagonism, suggesting that BcpA‐2 specifically recognizes *Burkholderia* GltJK. Using a series of chimeric proteins, the specificity determinant was mapped to *Burkholderia*‐specific sequences at the GltK C‐terminus, providing insight into BcpA transport across the recipient cell cytoplasmic membrane.

## Graphical abstract

Contact‐dependent growth inhibition systems mediate inter‐bacterial antagonism by delivering toxic effectors to neighboring bacteria. This study identifies inner membrane proteins GltJK as a likely receptor for recipient cell import of a *Burkholderia multivorans* CDI system effector, BcpA‐2. Specificity of the receptor is mapped to the GltK C‐terminus, suggesting that this region plays an important role in the translocation of BcpA‐2 across the recipient bacterium cytoplasmic membrane.

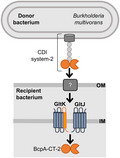

## 1 INTRODUCTION

Contact‐dependent growth inhibition (CDI) systems are Two‐Partner secretion systems that mediate competition and communication among closely related Gram‐negative bacteria. CDI systems are composed of a large stalk‐like exoprotein, termed BcpA in *Burkholderia* species, that is secreted to the cell surface by an outer membrane β‐barrel protein, BcpB. BcpA proteins have an N‐terminal conserved region approximately 2,700 amino acids (aa) in length that contains multiple filamentous hemagglutinin (FHA) repeats and a C‐terminal variable region (~300 aa) termed the BcpA‐CT (Anderson et al., 2012). This variable region contains an antibacterial toxic effector domain that frequently has nuclease activity (Nikolakakis et al., 2012). Upon cell−contact, the BcpA‐CT is delivered to the cytoplasm of neighboring bacteria where it inhibits growth unless the recipient cells produce a specific immunity protein, BcpI. BcpI proteins are also variable, only binding to and inhibiting the toxicity of their cognate BcpA‐CT proteins.

CDI systems were first described in *Escherichia coli* (where they are termed CdiBAI), and the pathway for effector entry into recipient cells has been delineated for several CdiA variants in *E. coli* and related species (Aoki et al., 2005; Ruhe et al., 2017; Willett et al., 2015). The current model suggests that a variable “receptor binding domain” of CdiA, mapped to ~300 aa in the conserved N‐terminal region between the FHA‐1 and FHA‐2 repeats and located distally from the donor cell surface, interacts with a specific recipient cell outer membrane protein (Ruhe et al., 2017, 2018). Upon binding, the CdiA‐CT domain is translocated across the recipient cell outer membrane. Recipient cell inner membrane proteins are required for subsequent CdiA‐CT translocation to the cytoplasm and sequences at the N‐terminal region of the CdiA‐CT (termed the “translocation domain”) dictate which inner membrane protein is required (Willett et al., 2015). Among others, outer membrane proteins BamA, OmpC, and Tsx and inner membrane proteins PtsG, MetI, RbsC, FtsH, and DppBC have been identified as receptors for CdiA variants (Allen et al., 2020; Ruhe et al., 2013, 2017; Willett et al., 2015).

The process of CdiA‐CT entry into recipient cells restricts effector exchange to bacterial species that produce the required receptors. Further specificity has also been observed for the translocation of several CdiA‐CT polypeptides, due to sequence variability in the outer membrane proteins that serve as CdiA receptors. For example, CdiA proteins that utilize BamA as an outer membrane receptor are constrained by strain‐specific differences in the variable extracellular loops of BamA (Ruhe et al., 2013). Thus, although BamA is generally conserved among enterobacteria, these CdiA proteins are only delivered to bacteria that produce the “correct” BamA variant. It is not known whether similar sequence specificity may exist for CdiA inner membrane receptors.

*Burkholderia* CDI systems are functionally distinct from the CdiBAI proteins found in *E. coli* and related species. Differences include gene order (*cdiBAI* for “*E. coli‐*type,” *bcpAIOB* for “*Burkholderia*‐type”), the motif that separates the conserved CdiA/BcpA N‐terminal region from the variable C‐terminal toxin (VENN in *E. coli*, N(E/Q) × LYN in *Burkholderia*), the modularity of CdiA/BcpA proteins, and the presence of one or more open reading frames between *bcpI* and *bcpB*, termed *bcpO* genes (Anderson et al., 2012; Nikolakakis et al., 2012). Evidence suggests that the BcpA‐CT recipient cell entry pathway is similar to pathways described for CdiA‐CT proteins, but only one BcpA receptor, an inner membrane protein, has been conclusively identified (Koskiniemi et al., 2015; Willett et al., 2015).

We recently described the two CDI systems of *Burkholderia cepacia* complex (Bcc) strain *Burkholderia multivorans* CGD2M (Myers‐Morales et al., 2019). Like other *Burkholderia* species, Bcc organisms are commonly found in soil and other natural environments (Vial et al., 2010). They are also opportunistic pathogens that can cause chronic pulmonary infections in patients with cystic fibrosis, chronic granulomatous disease, or who are immunocompromised. Here we used a transposon mutagenesis approach to define factors in *B. multivorans* recipient cells that are required for intoxication by BcpA and we identified GltJ/GltK as the likely inner membrane receptors for BcpA‐2. Strikingly, although *B. multivorans* and *E. coli* GltJK share >60% sequence identity, only the *Burkholderia* homologs conferred susceptibility to BcpA‐2. Using a series of chimeric proteins, this specificity was mapped to the C‐terminus of GltK. Containing predicted periplasmic, transmembrane, and cytoplasmic sequences, our results support a model in which this GltK region is critical for translocation of BcpA‐2‐CT across the recipient cell cytoplasmic membrane.

## 2 RESULTS

### 2.1 Transposon insertions in *gltJK* confer resistance to BcpA‐2

**FIGURE 1:**
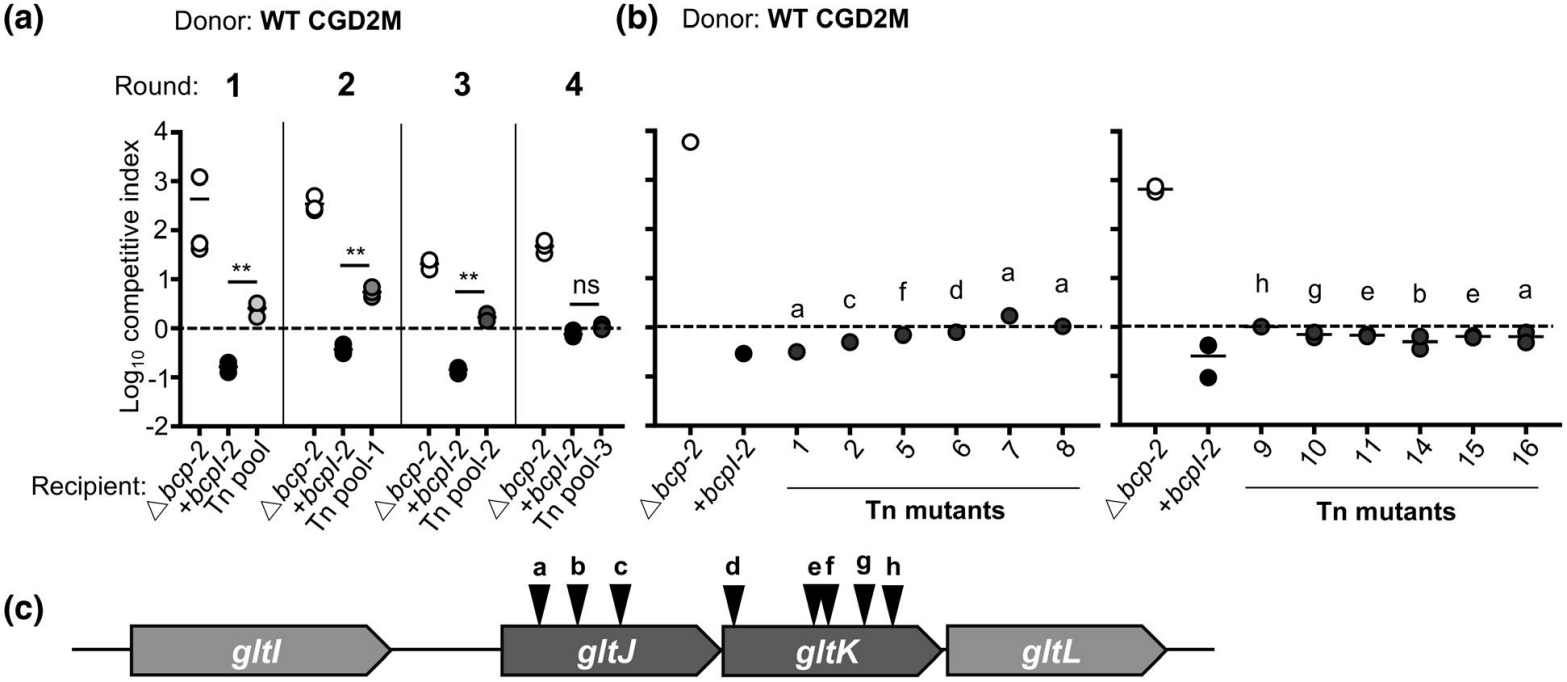
Transposon mutagenesis and selection for contact‐dependent growth inhibition (CDI)‐resistant *Burkholderia multivorans* mutants. (a) *B. multivorans* CGD2M Δ*bcp‐2* recipient bacteria were mutagenized with miniTn5, pooled, and subjected to sequential rounds of co‐culture with wild‐type bacteria on agar. Competitive index (CI) values are shown for competition mixtures collected at 24 hr for Cm^R^ wild‐type donor bacteria co‐cultured with Kan^R^ Δ*bcp‐2* recipient bacteria (none; open symbols), Δ*bcp‐2* recipients complemented with cognate *bcpI‐2* (black symbols), and the Δ*bcp‐2* transposon (Tn) mutant pool (gray symbols). Output CFUs for the Δ*bcp‐2* transposon mutant pool were collected after each competitive round, pooled, and used as the input for the next round. The CI was calculated as (output donor CFU/recipient CFU) divided by (input donor CFU/recipient CFU). Symbols represent CI values from one biological replicate and bars show the mean (*n* = 3). Dashed line shows CI = 1 (1:1 ratio of donor:recipient). CI values for the Tn mutant pool were compared to those of immune recipient bacteria (+*bcpI‐2*). Statistical differences were determined with two‐tailed Student’s *t*‐test. **p < .01. ns, not significant. (b) Competitive indices for wild‐type donor bacteria co‐cultured with Δ*bcp‐2* recipient bacteria (none; open symbols), Δ*bcp‐2* recipients complemented with cognate *bcpI‐2*, and individual Δ*bcp‐2* transposon mutants. Individual mutants were tested in two batches (left and right panels). Letters (a‒h) indicate the location of the transposon insertion for each mutant in panel C locus diagram. (c) Diagram showing the genetic organization *B. multivorans* CGD2M *gltIJKL* locus. Arrows indicate the approximate locations of unique transposon insertions conferring resistance to BcpA‐2‐mediated CDI (letters correspond to Tn mutants in b)

*B. multivorans* CGD2M encodes two distinct functional CDI systems, but only CDI system‐2 mediates interbacterial competition in laboratory medium under conditions of native gene expression (Myers‐Morales et al., 2019). To determine factors needed for translocation and toxicity of BcpA‐2 in *B. multivorans* recipient cells, we used a sequential selection approach similar to approaches used to analyze *E. coli* CdiBAI (Aoki et al., 2008; Willett et al., 2015). The CDI‐sensitive Δ*bcpAIB‐2* mutant was mutagenized with miniTn5 and the resulting mutant library competed against wild‐type *B. multivorans* in an interbacterial competition assay (Figure 1a and Table S1). Transposon mutants surviving the competition were pooled and re‐competed against wild‐type bacteria to enrich for insertions that conferred resistance to BcpA‐2. This process was repeated until, after four sequential rounds of competition, CDI susceptibility of the enriched transposon mutant pool was not significantly different from that of recipient cells expressing cognate *bcpI‐2*. CDI resistance was validated for 16 randomly chosen clones by testing them in individual competition assays against wild‐type bacteria (Figure 1b). Using nested arbitrary primed PCR, transposon insertion sites were identified for 12 of these mutants. All 12 mutants had insertions in *gltJ* (3 unique insertions) or *gltK* (5 unique insertions; Table S2). Predicted to encode inner membrane components of an ABC‐type transporter for glutamate and aspartate, *gltJK* are found in a locus that also includes *gltL*, encoding a predicted ATPase, and *gltI*, encoding a predicted periplasmic binding protein (Figure 1c).

### 2.2 GltJK are required for recipient cell sensitivity to BcpA‐2

**FIGURE 2:**
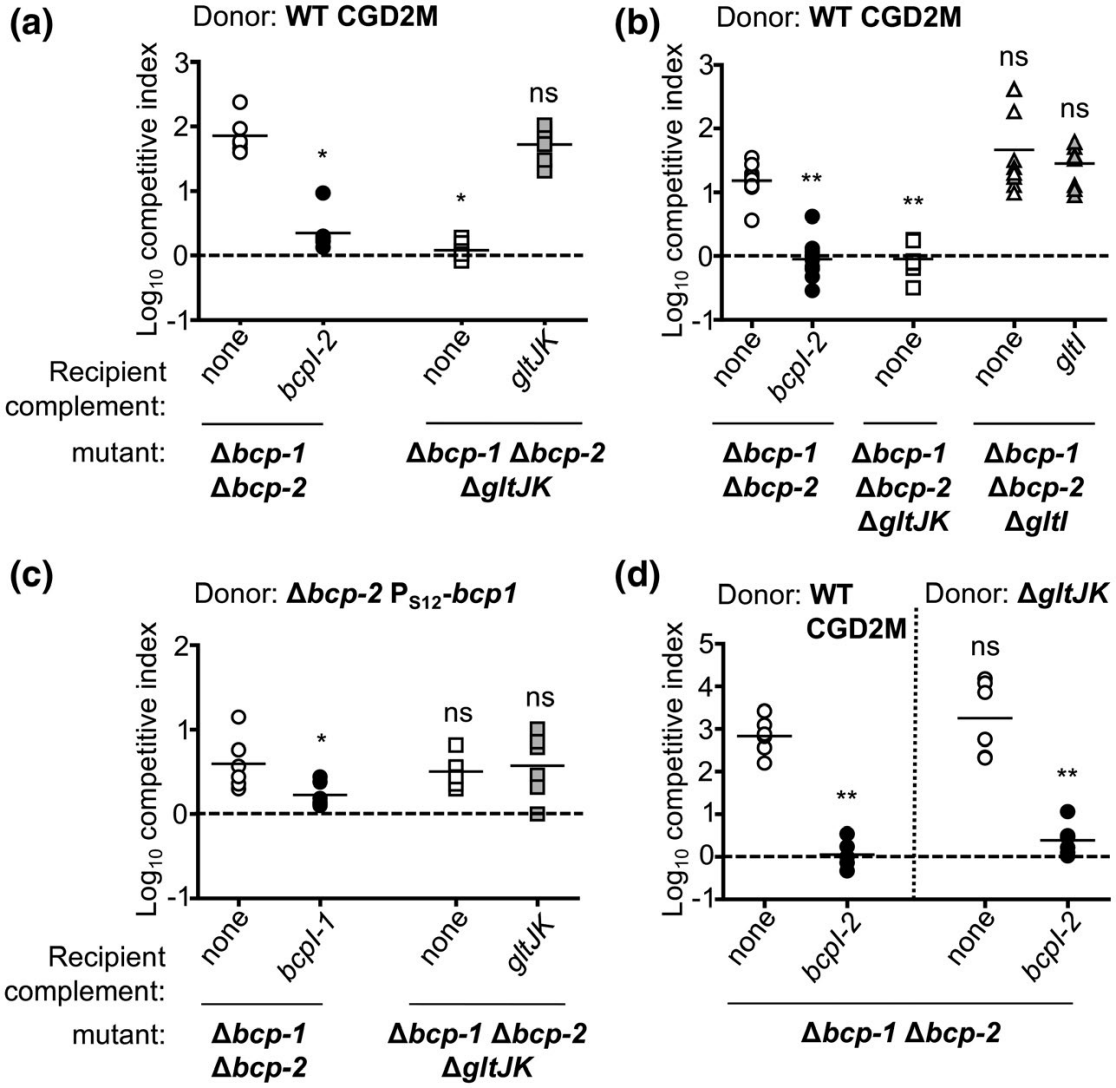
Roles of *gltJ*, *gltK* and *gltI* in recipient cell susceptibility to *Burkholderia multivorans* contact‐dependent growth inhibition. Competitive indices (CI) for bacteria sampled from the edge of a colony biofilm after 24 hr co‐culture on agar are shown. CI was calculated as (output donor CFU/recipient CFU) divided by (input donor CFU/recipient CFU). Symbols represent CI from one biological replicate, and bars show the mean (*n* = 6 from two independent experiments). Dashed line shows CI = 1 (no competition). Statistical differences were determined with two‐tailed Student’s *t*‐test unless otherwise indicated. *p < .05; **p < .01; compared to corresponding recipient cells with no *bcpI*. (a) Wild‐type donor bacteria were competed against Δ*bcp‐1* Δ*bcp‐2* recipient cells (open circles), Δ*bcp‐1* Δ*bcp‐2* recipients carrying *bcpI‐2* at an attTn7 site (black circles), and Δ*bcp‐1* Δ*bcp‐2* Δ*gltJK* recipient cells with (gray squares) or without (white squares) *gltJK* at an *att*Tn7 site. Statistical differences were determined with the Mann−Whitney test. (b) Wild‐type donor bacteria were competed against mutants in a) and *bcp‐1* Δ*bcp‐2* Δ*gltI* recipient cells with (gray triangles) or without (white triangles) *gltI* at an *att*Tn7 site. Statistical differences were determined with the Mann−Whitney test. (c) *B. multivorans* Δ*bcp‐2* donor bacteria overexpressing *bcpAIOB‐1* (from strong, constitutive promoter P_S12_) were competed against Δ*bcp‐1* Δ*bcp‐2* recipient cells (open circles), Δ*bcp‐1* Δ*bcp‐2* recipients carrying *bcpI‐1* at an attTn7 site (black circles), and Δ*bcp‐1* Δ*bcp‐2* Δ*gltJK* recipient cells with (gray squares) or without (white squares) *gltJK* at an *att*Tn7 site. (d) Wild‐type (left panel) or Δ*gltJK* (right panel) donor bacteria were competed against Δ*bcp‐1* Δ*bcp‐2* recipient cells with (black circles) or without (white circles) cognate *bcpI‐2* at an *att*Tn7 site

To test the role of *gltJK* in *B. multivorans* CDI, we constructed a Δ*gltJK* deletion mutation in a Δ*bcp‐1*Δ*bcp‐2* double mutant that lacks both of its CDI systems. This quadruple mutant was resistant to interbacterial competition with wild‐type bacteria and its sensitivity was restored by complementation of *gltJK* to a chromosomal site (Figure 2a). During culture in rich medium, the second *B. multivorans* CDI system, BcpAIOB‐1, mediates a lower level of interbacterial competition than BcpAIB‐2 and overexpression of *bcpAIOB‐1* is necessary to observe its effects in vitro (Myers‐Morales et al., 2019). To determine whether *gltJK* are also required for BcpA‐1 susceptibility, the quadruple Δ*gltJK* Δ*bcp‐1*Δ*bcp‐2* mutant was competed against a Δ*bcp‐2* mutant that overexpressed *bcpAIOB‐1*. No significant difference in competitive index was seen between recipient cells with and without *gltJK* (Figure 2c), indicating that GltJK are not required for BcpA‐1 sensitivity and are specifically required for *B. multivorans* BcpA‐2. To determine whether loss of *gltJK* in donor bacteria alters CDI, we constructed a Δ*gltJK* mutant in wild‐type *B. multivorans*. Loss of *gltJK* had no effect on the ability of this mutant to inhibit the growth of recipient cells (Figure 2d), indicating that *gltJK* are not required in donor bacteria for efficient CDI.

ABC‐type transporters like GltJK have been previously found to mediate translocation of CdiA‐CT effectors into recipient cells (Willett et al., 2015). The ATPase components of these transporters, including GltL, were not required for CdiA translocation, but the periplasmic components of the transporters have not been investigated in the context of CDI (Willett et al., 2015). To determine whether the predicted periplasmic binding protein GltI influences BcpA‐2 sensitivity, a Δ*gltI* mutant was constructed in a Δ*bcp‐1*Δ*bcp‐2* double mutant. This mutant showed no difference in interbacterial competition against wild‐type *B. multivorans* (Figure 2b), indicating that *gltI* does not contribute to CDI susceptibility. Although the ATPase component (GltL) was not examined here, these data are consistent with the model that recipient cell sensitivity to BcpA‐2 requires the membrane‐localized components of the GltIJKL ABC‐type transporter.

### 2.3 GltJK are not required for BcpA‐2 intracellular toxicity

**FIGURE 3:**
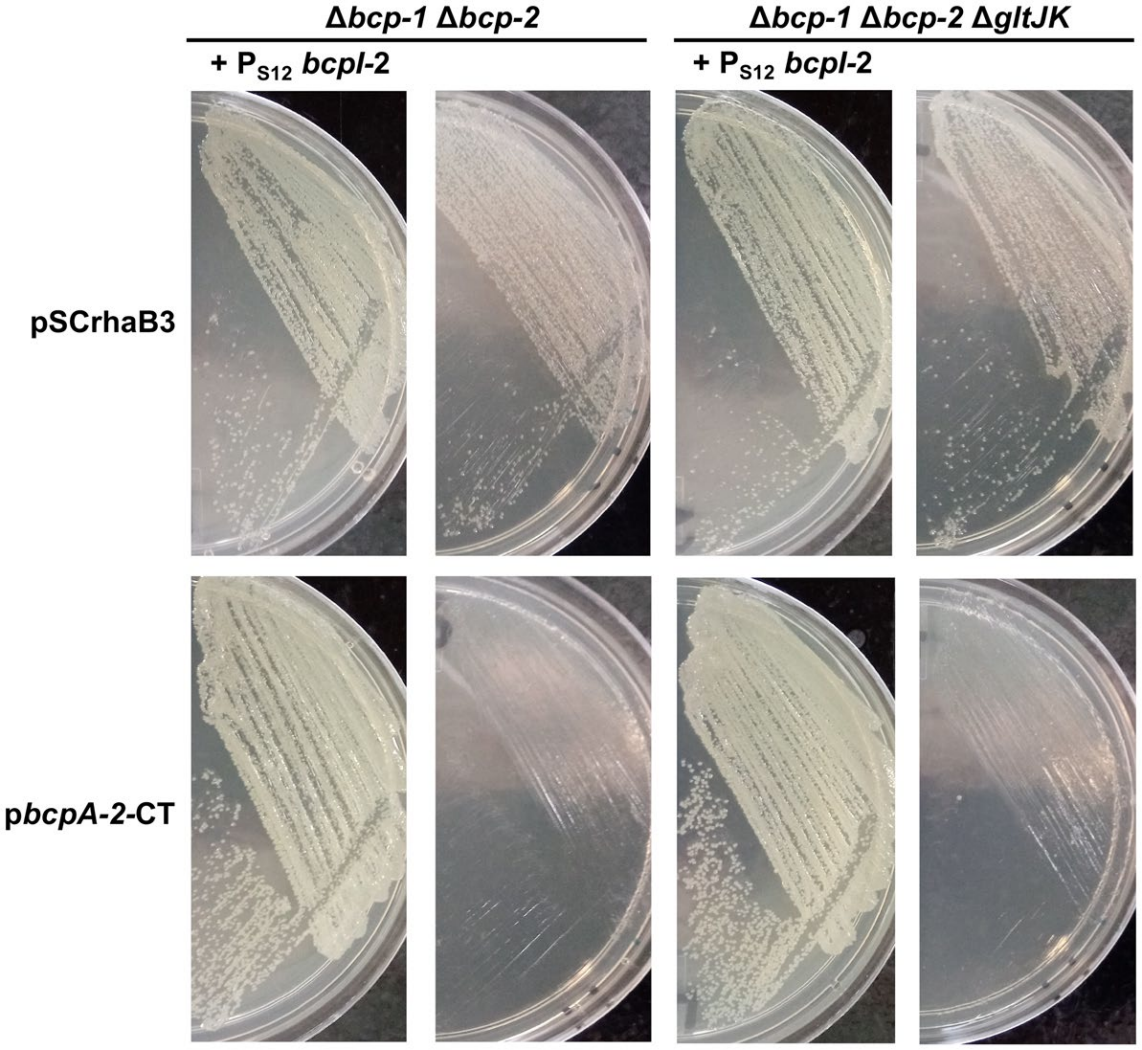
Role of *gltJK* in BcpA‐2‐CT intracellular toxicity. Transconjugants obtained after introduction of pSCrhaB3 empty vector (top) or plasmid carrying *bcpA‐2‐CT* (bottom) into Δ*bcp‐1* Δ*bcp‐2* mutant bacteria (left two columns) or Δ*bcp‐1* Δ*bcp‐2* Δ*gltJK* mutant bacteria (right two columns) that constitutively express cognate *bcpI‐2* from an *att*Tn7 site (first and third columns) or lack immunity (second and fourth columns)

Predicted to be located in the inner membrane, GltJK are hypothesized to impact BcpA‐2 translocation to the recipient cell cytoplasm. To test this hypothesis, we examined the intracellular toxicity of BcpA‐2‐CT independently of effector translocation. A plasmid containing *bcpA‐2‐CT* under the control of a rhamnose‐inducible promoter was constructed and introduced into Δ*bcp‐1*Δ*bcp‐2* and Δ*bcp‐1*Δ*bcp‐2* Δ*gltJK* recipient cells by conjugation. Upon selection on medium containing glucose (allowing only basal *bcpA‐2‐CT* expression), *B. multivorans* transconjugants could not be obtained for either *gltJK*+ or *gltJK*− recipient bacteria, unless bacteria were complemented with *bcpI‐2* (Figure 3). Thus, when BcpA‐2 entry into recipient cells was bypassed by producing BcpA‐2‐CT intracellularly, cells with and without GltJK were similarly susceptible to toxicity. These results indicate that GltJK are not required for BcpA‐2 intracellular toxicity and instead suggest that GltJK are needed for BcpA‐2 translocation into recipient cells, likely across the cytoplasmic membrane.

### 2.4 *E*. *coli gltJK* cannot complement *B. multivorans* Δ*gltJK*

**FIGURE 4:**
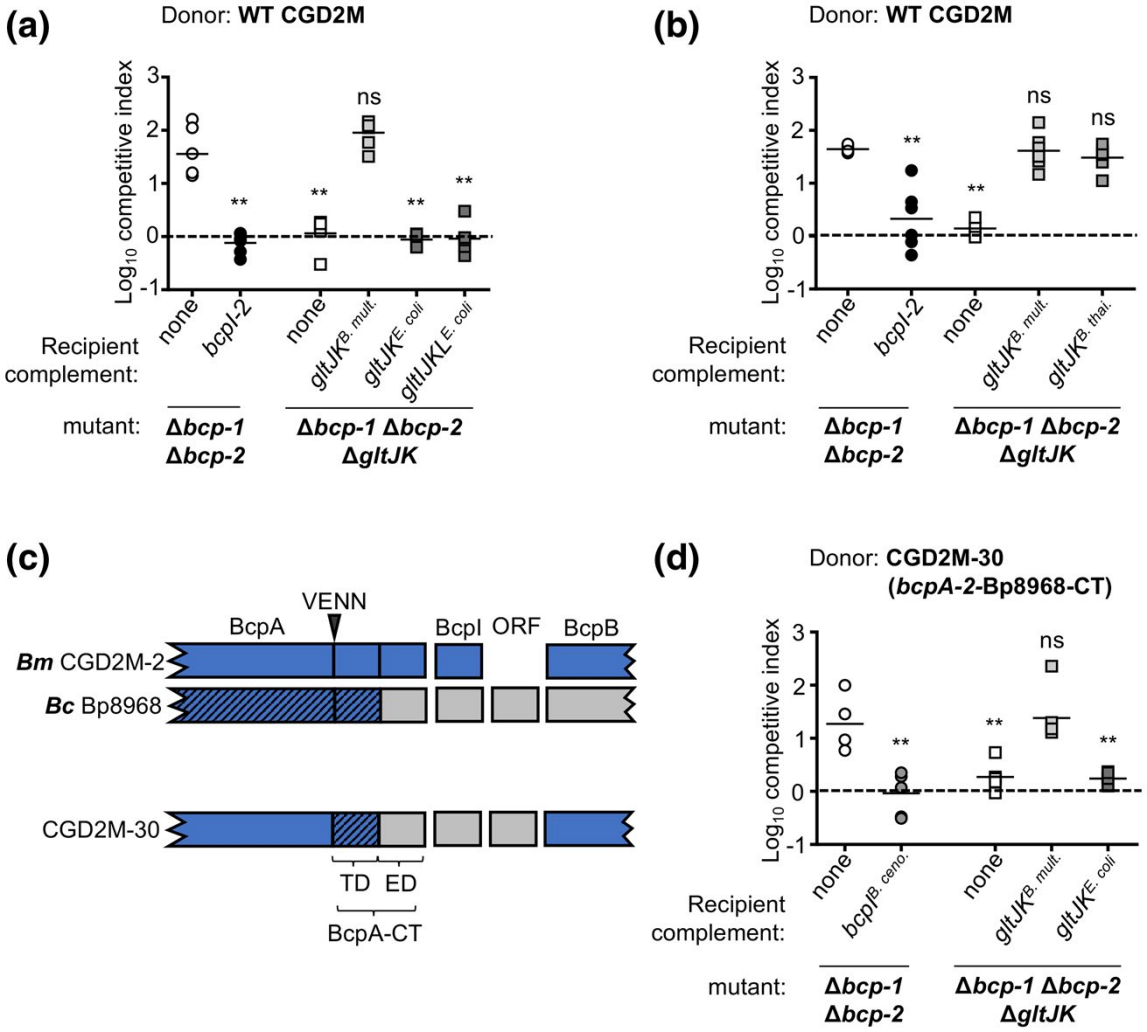
Ability of heterologous *gltJK* alleles to sensitize *Burkholderia multivorans* recipient cells to contact‐dependent growth inhibition mediated by BcpA‐2 and a related BcpA protein. Competitive indices (CI) for bacteria sampled from the edge of a colony biofilm after 24 hr co‐culture on agar are shown. CI was calculated as (output donor CFU/recipient CFU) divided by (input donor CFU/recipient CFU). Symbols represent CI from one biological replicate and bars show the mean (*n* = 6 from two independent experiments). Dashed line shows CI = 1 (no competition). Statistical differences were determined with Mann−Whitney test unless otherwise indicated. **p < .01; compared to corresponding recipient cells with no *bcpI*. (a) Wild‐type donor bacteria were competed against Δ*bcp‐1* Δ*bcp‐2* recipient cells (open circles), Δ*bcp‐1* Δ*bcp‐2* recipients carrying *bcpI‐2* at an *att*Tn7 site (black circles), and Δ*bcp‐1* Δ*bcp‐2* Δ*gltJK* recipient cells complemented at an *att*Tn7 site with empty vector (none, white squares), or P_S12_‐driven *B. multivorans gltJK* (gray squares), *Escherichia coli gltJK* (dark gray squares) or *E. coli gltIJKL* (dark gray squares). (b) Wild‐type donor bacteria were competed against Δ*bcp‐1* Δ*bcp‐2* recipient cells (open circles), Δ*bcp‐1* Δ*bcp‐2* recipients carrying *bcpI‐2* at an *att*Tn7 site (black circles), and Δ*bcp‐1* Δ*bcp‐2* Δ*gltJK* recipient cells complemented at an *att*Tn7 site with empty vector (none, white squares) or P_S12_‐driven *B. multivorans gltJK* (gray squares) or *Burkholderia thailandensis gltJK* (dark gray squares). Statistical differences were determined with two‐tailed Student’s *t*‐test. (c) Graphical representations of the BcpA‐CT domains and BcpI proteins from *B. multivorans* CGD2M (top), *Burkholderia cenocepacia* Bp8968 (middle) and chimeric strain constructed (CGD2M‐30, bottom). Arrow indicates the location of the VENN motif and theoretical N‐terminal end of the BcpA‐CT region. Approximate locations of the putative translocation domain (TD) and effector domain (ED) of the BcpA‐CT are shown. (d) Chimeric *B. multivorans* donor bacteria (CGD2M‐30) producing chimeric BcpA‐2 containing the BcpA‐CT from *B. cenocepacia* Bp8968 (along with its cognate BcpI and an additional hypothetical ORF) was competed against Δ*bcp‐1* Δ*bcp‐2* recipient cells (open circles), Δ*bcp‐1* Δ*bcp‐2* recipients carrying *B. cenocepacia* Bp8968 *bcpI* at an *att*Tn7 site (gray circles), and Δ*bcp‐1* Δ*bcp‐2* Δ*gltJK* recipient cells complemented at an *att*Tn7 site with empty vector (none, white squares), or P_S12_‐driven *B. multivorans gltJK* (gray squares) or *E. coli gltJK* (dark gray squares)

Previous research has already identified *E. coli* GltJK as a probable cytoplasmic membrane translocation factor for CdiA‐CT from *Photorhabdus luminescens* strain TTO1 (Willett et al., 2015). CdiA‐CT proteins that require the same translocation factor have been shown to share a high degree of sequence identity within their N‐terminal regions, termed the “translocation domain” (Willett et al., 2015). Unexpectedly, comparison of the GltJK‐utilizing BcpA‐2‐CT^CGD2M^ and CdiA‐CT^TTO1^ showed only 24% sequence identity within the N‐terminal halves of the polypeptides (Figure S1). By contrast, GltJ and GltK are more similar between *B. multivorans* and *E. coli*, sharing 63% and 71% amino acid identity, respectively (78% and 84% similarity, respectively). To explore the impact of these differences on BcpA‐2 uptake, *E. coli* DH5α *gltJK* (*gltJK^E. coli^*) were expressed in the Δ*bcp‐1*Δ*bcp‐2* Δ*gltJK* quadruple mutant. In interbacterial competition assays, *E. coli gltJK* failed to restore CDI sensitivity of this mutant (Figure 4a). In the event that the *E. coli* GltJK proteins require the cognate *E. coli* GltI and GltL proteins for optimal function or stability, we also attempted to complement the mutant with the complete *gltIJKL* locus from *E. coli* (*gltIJKL^E. coli^*). Expression of these genes was also unable to restore BcpA‐2 sensitivity to recipient cells lacking *gltJK* (Figure 4a). *Burkholderia thailandensis* E264 GltJK are very similar to their *B. multivorans* CGD2M counterparts (98% and 96% identical, respectively) and, as expected, the genes encoding these proteins (*gltJK^B. thai^*) also restored susceptibility of the *B. multivorans* recipient cells to BcpA‐2 toxicity (Figure 4b). These results suggest that CGD2M BcpA‐2 requires *Burkholderia* GltJK for translocation and led us to hypothesize that specific differences in the *E. coli* proteins block entry of BcpA‐2 into the recipient cell.

### 2.5 Related BcpA‐CT shows specificity for *Burkholderia* GltJK

Bioinformatic analyses of other *Burkholderia* CDI systems and comparison of the predicted BcpA‐CT translocation domains led to the identification of a putative BcpA from *Burkholderia cenocepacia* Bp8968. BcpA‐CT^Bp8968^ and BcpA‐2‐CT^CGD2M^ share 91% identity across their N‐terminal regions (~150 residues), but only 14% identity over the C‐terminal halves of BcpA‐CT (Figure S1). The large, conserved N‐termini (~3,000 residues) of the two BcpA proteins similarly share high sequence identity (93%). To test the hypothesis that BcpA‐CT^Bp8968^ would also utilize GltJK for entry into recipient cells, we constructed a chimeric mutant by replacing the chromosomal *B. multivorans bcpA‐2‐CT* and *bcpI* with *B. cenocepacia* Bp8968 *bcpA‐CT* and *bcpI* (Figure 4c). An additional predicted *B. cenocepacia* Bp8968 ORF (DF018_16545) located between *bcpI* and *bcpB* was also included in the chimeric mutant. This mutant (named CGD2M‐30), expected to produce chimeric BcpA along with cognate BcpI, was used as a donor in competition assays. CGD2M‐30 outcompeted *B. multivorans* Δ*bcp‐1*Δ*bcp‐2* recipient cells (Figure 4d) and Δ*bcp‐2* recipient cells (Figure S2a) by ~1.5 logs unless they produced cognate *B. cenocepacia bcpI*, indicating that the chimeric BcpA was functional. As expected, this mutant was unable to inhibit the growth of recipient cells lacking *gltJK* or expressing *E. coli gltJK* (Figures 4d and S2a), supporting the results obtained with native BcpA‐2. Although we cannot rule out contributions of the conserved BcpA N‐terminus (~3,000 aa), these results are consistent with previous data suggesting that the BcpA‐CT N‐terminal region functions in toxin translocation. These findings are also consistent with our hypothesis that the translocation domain found in BcpA‐2‐CT^CGD2M^ and BcpA‐CT^Bp8968^ is specific for *Burkholderia* GltJK.

### 2.6 BcpA^CGD2M^‐CdiA^TTO1^ chimera is not functional

Unlike *E. coli* CdiA proteins, previous data suggest that the N‐terminal “conserved” region of *Burkholderia* BcpA proteins is only capable of delivering certain, closely related BcpA‐CT toxin cargo (from BcpA proteins of the same subclass) (Anderson et al., 2014; Nikolakakis et al., 2012). Chimeric proteins consisting of “mismatched” BcpA N‐ and C‐terminal regions are nonfunctional for CDI (Anderson et al., 2014). Nonetheless, to further test our hypothesis, we attempted to switch the GltJK specificity of *B*. *multivorans* BcpA‐2 by generating a chimeric BcpA protein composed of the BcpA‐2 N‐terminus (~3,000 aa) and the *P*. *luminescens* TTO1 CdiA‐CT, joined at the VENN motif (Figure S2b). A *Burkholderia* mutant producing this chimeric protein and its cognate immunity protein, CdiI^TTO1^, did not inhibit the growth of *B. multivorans* recipient cells expressing either *B. multivorans* or *E. coli gltJK* (Figure S2). These results suggest that *E. coli* GltJK are insufficient for CdiA‐CT^TTO1^ translocation/toxicity in *B. multivorans* or, more likely, that this chimeric BcpA^CGD2M^‐CdiA^TTO1^ protein is not functional. While we cannot rule out the possibility here that *E. coli* GltJK are improperly assembled in *B. multivorans* and not competent for CdiA‐CT^TTO1^ translocation, previous evidence indicates that *Burkholderia* BcpA proteins are not as modular as their CdiA counterparts, which can complicate generation of functional chimeras (Anderson et al., 2014).

### 2.7 Mixed GltJK complexes suggest GltK drives translocation specificity

**FIGURE 5:**
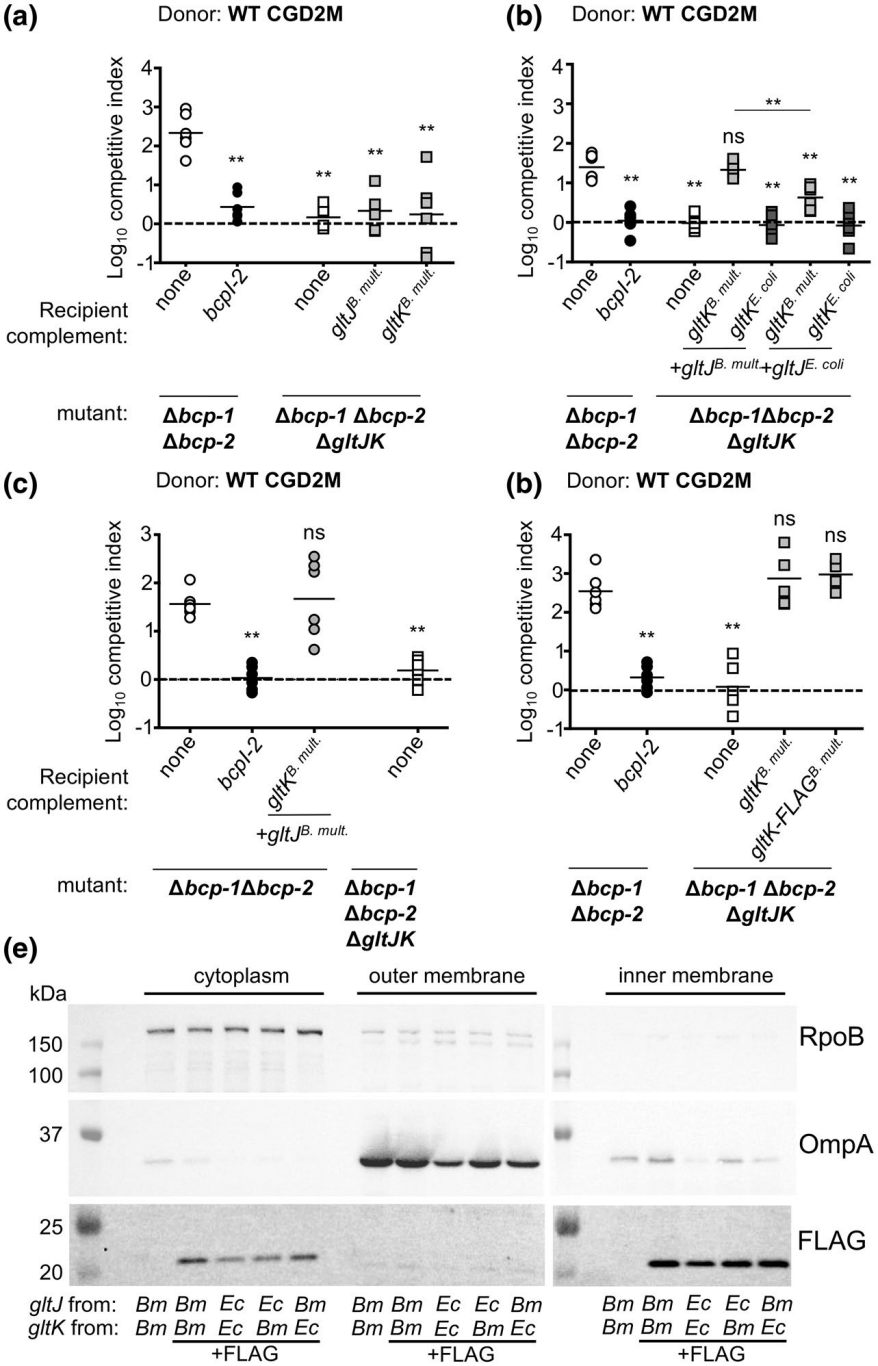
Individual contributions of GltJ and GltK to sensitivity to BcpA‐2‐mediated contact‐dependent growth inhibition. (a) Wild‐type donor bacteria were competed against Δ*bcp‐1* Δ*bcp‐2* recipient cells (open circles), Δ*bcp‐1* Δ*bcp‐2* recipients carrying *bcpI‐2* at an *att*Tn7 site (black circles), and Δ*bcp‐1* Δ*bcp‐2* Δ*gltJK* recipient cells complemented at an *att*Tn7 site with empty vector (none, white squares) or P_S12_‐driven *Burkholderia multivorans gltJ* or *gltK* (gray squares). Competitive indices (CI) for bacteria sampled from the edge of a colony biofilm after 24 hr co‐culture on agar are shown. CI was calculated as (output donor CFU/recipient CFU) divided by (input donor CFU/recipient CFU). Symbols represent CI from one biological replicate and bars show the mean (*n* = 6 from two independent experiments). Dashed line shows CI = 1 (no competition). Statistical differences were determined with two‐tailed Student’s *t*‐test. **p < .01; compared to corresponding recipient cells with no *bcpI* unless otherwise indicated. (b) Competition assays as described in a. Wild‐type donor bacteria were also competed against Δ*bcp‐1* Δ*bcp‐2* Δ*gltJK* recipient cells complemented at each of two *att*Tn7 sites with combinations of P_S12_‐driven *gltJ* or *gltK* from *Escherichia coli* or *B. multivorans*. (c) Competition assays as described in a. Wild‐type donor bacteria were also competed against Δ*bcp‐1* Δ*bcp‐2* recipient cells complemented at each of two *att*Tn7 sites with *B. multivorans gltJ* and *gltK* (gray circles). (d) Competition assays as described in a. Wild‐type donor bacteria were also competed against Δ*bcp‐1* Δ*bcp‐2* Δ*gltJK* recipient cells complemented at each of two *att*Tn7 sites with empty vectors (none, white squares) or *B. multivorans gltJ* and untagged *gltK* or FLAG‐tagged *gltK* (gray squares). (e) Western blots of subcellular fractions of Δ*bcp‐1* Δ*bcp‐2* Δ*gltJK* bacteria complemented at each of two *att*Tn7 sites with combinations of *gltJ* and FLAG‐tagged *gltK* from *B. multivorans* and *E. coli* (or untagged *gltK*; first lanes of each fraction). Equal amounts of each fraction (cytoplasmic, outer membrane [Sarkosyl‐soluble], and inner membrane [Sarkosyl‐insoluble]) were resolved on 12% sodium dodecyl sulfate‐polyacrylamide gel electrophoresis gels and blots probed with anti‐ FLAG peptide (bottom), anti‐*E. coli* RNA Polymerase β subunit (RpoB; top), and anti‐*Salmonella typhii* Outer Membrane protein A (OmpA, middle) antibodies. Expected masses for GltK, OmpA and RpoB are ~26, ~35, and 150 kDa, respectively

To determine how each inner membrane protein contributes to BcpA‐2 translocation, we generated plasmids to deliver individual *gltJ* and *gltK* genes to each of the *B. multivorans* two *att*Tn7 sites. Neither *gltJ* nor *gltK* expressed alone was sufficient to restore CDI susceptibility to recipient cells lacking *gltJK* (Figure 5a), suggesting that a membrane complex composed of both GltJ and GltK are needed for BcpA‐CT translocation and/or complex stability. Growth inhibition also did not occur when recipient cells expressed *E. coli gltK* paired with *gltJ* from either *E. coli* or *B. multivorans* (Figure 5b). Surprisingly, *E. coli gltJ* expressed with *B. multivorans gltK* conferred partial susceptibility to BcpA‐2, although the level of growth inhibition was significantly less than when both *gltJ* and *gltK* were from *B. multivorans* (Figure 5b). Overexpression of *gltJK* in recipient cells that expressed native *gltIJKL* did not enhance CDI susceptibility (Figures 5c and S4a). Together, these data indicate that both GtlJ and GltK are required for BcpA‐2 import into recipient cells, but suggest that GltK confers most of the species specificity to translocation.

It is possible that *E. coli* GltJK produced heterologously in *B. multivorans* results in unstable, improperly folded, or incorrectly localized proteins. To determine whether the observed differences in CDI susceptibility were due to improper localization of *E. coli* GltK when produced in *B. multivorans*, we generated epitope‐tagged versions of each GltK that carried an in‐frame FLAG tag at its C‐terminus. The FLAG epitope tag did not appear to disrupt the function of *B. multivorans* GltK, as there was no difference in CDI susceptibility between recipient cells producing tagged or untagged *B. multivorans* GltK (Figure 5d).

Localization of GltK‐FLAG in these recipient cells was determined by subcellular fractionation using Sarkosyl solubility methods (Filip et al., 1973; Myers‐Morales et al., 2007). As controls, fractions were probed with anti‐*E. coli* RpoB antibodies, which recognize a cytoplasmic *B. multivorans* protein that is a similar size to the *E. coli* 153 kDa RpoB (Figure S3). Fractions were also probed with anti‐*Salmonella* OmpA antibodies, which recognize an outer membrane *B. multivorans* protein that is a similar size to the 35 kDa *E. coli* OmpA (Figure S3). Bands reacting with anti‐RpoB were observed primarily in the cytoplasmic fraction, with very faint bands observed in the membrane fractions (Figure 5e), suggesting little cytoplasmic contamination of the membrane fractions. Similarly, anti‐OmpA‐reactive bands were localized to the outer membrane (Sarkosyl‐insoluble) fraction, with a small amount of putative OmpA protein observed in the inner membrane (Sarkosyl‐soluble) fractions.

For all mutants, GltK*^E. coli^*‐FLAG and GltK*^B. multi^*‐FLAG were detected in the inner membrane‐enriched fractions, with fainter bands detectable in the cytoplasmic fractions, suggesting that all GltK proteins, regardless of their species or GltJ partner, were localized to the *B. multivorans* inner membrane (Figures 5e and S5). Even in the absence of GltJ, GltK*^B. multi^*‐FLAG was detectable in the inner membrane fraction (Figure S4b,c). Both GltK*^E. coli^*‐FLAG and GltK*^B. multi^*‐FLAG ran at a slightly smaller molecular weight than predicted. Slight differences in GltK‐FLAG levels were detectable, with GltK*^B. multi^*‐FLAG appearing to be the most abundant when produced with its cognate GltJ*^B. multi^* protein. Inadequate levels of GltK*^E. coli^* could affect BcpA‐2‐CT translocation in a non‐specific manner. However, GltK‐FLAG abundance in the inner membrane (Figure 5e) did not appear to correlate with BcpA‐2 susceptibility (Figure 5b). These results indicate that both GltK*^B .multi^* and GltK*^E. coli^* localize to the *B. multivorans* inner membrane fraction and suggest that these proteins differ in their ability to mediate BcpA‐2‐CT translocation.

### 2.8 Multiple regions of GltJ are required for BcpA‐2 translocation specificity

**FIGURE 6:**
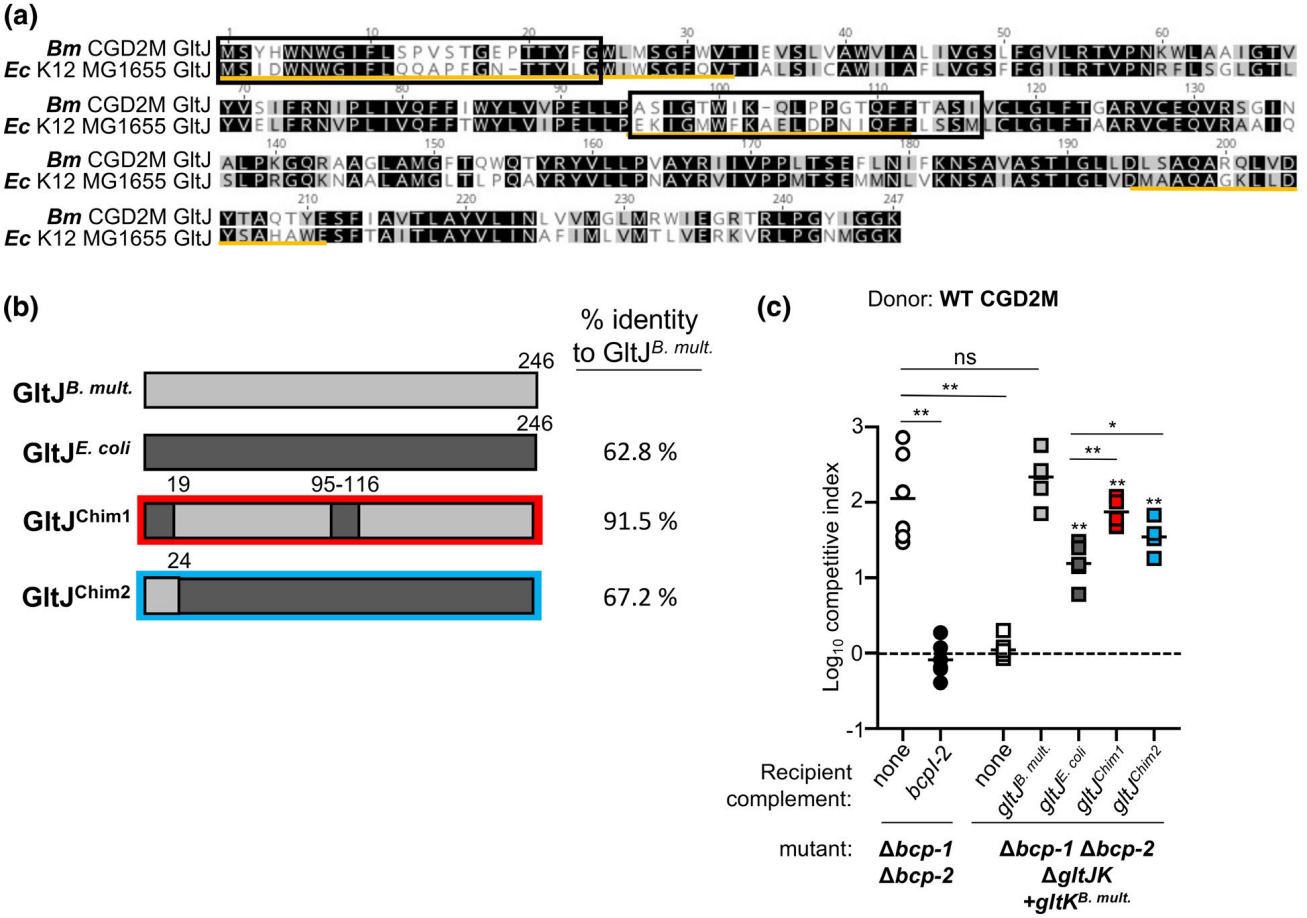
Contribution of GltJ to the specificity of BcpA‐2 translocation. (a) Amino acid alignment of GltJ from *Burkholderia multivorans* CGD2M and *Escherichia coli* MG1655. Yellow lines indicate the predicted periplasmic regions and black boxes show the regions changed in the constructed chimeras. (b) Graphic representation of the GltJ proteins from *B. multivorans* and *E. coli* and the chimeras constructed with indicated residue numbers. Percent identity of each protein/chimera to *B. multivorans* CGD2M GltJ is shown to the right. (c) Wild‐type donor bacteria were competed against Δ*bcp‐1* Δ*bcp‐2* recipient cells (open circles), Δ*bcp‐1* Δ*bcp‐2* recipients carrying *bcpI‐2* at an *att*Tn7 site (black circles), and Δ*bcp‐1* Δ*bcp‐2* Δ*gltJK* recipient cells complemented at each of two *att*Tn7 sites with empty vectors (none, white squares) or P_S12_‐driven *B. multivorans gltK* and the indicated *gltJ* allele: *B*. *multivorans gltJ* (gray squares), *E. coli gltJ* (dark gray squares), or genes to produce chimeric GltJ proteins *gltJ^Chim1^* (red squares) or *gltJ^Chim2^* (blue squares). Competitive indices (CI) for bacteria sampled from the edge of a colony biofilm after 24 hr co‐culture on agar are shown. CI was calculated as (output donor CFU/recipient CFU) divided by (input donor CFU/recipient CFU). Symbols represent CI from one biological replicate and bars show the mean (*n* = 6 from two independent experiments). Dashed line shows CI = 1 (no competition). Statistical differences were determined with two‐tailed Student’s *t*‐test. *p < .05; **p < .01; compared as shown

**FIGURE 7:**
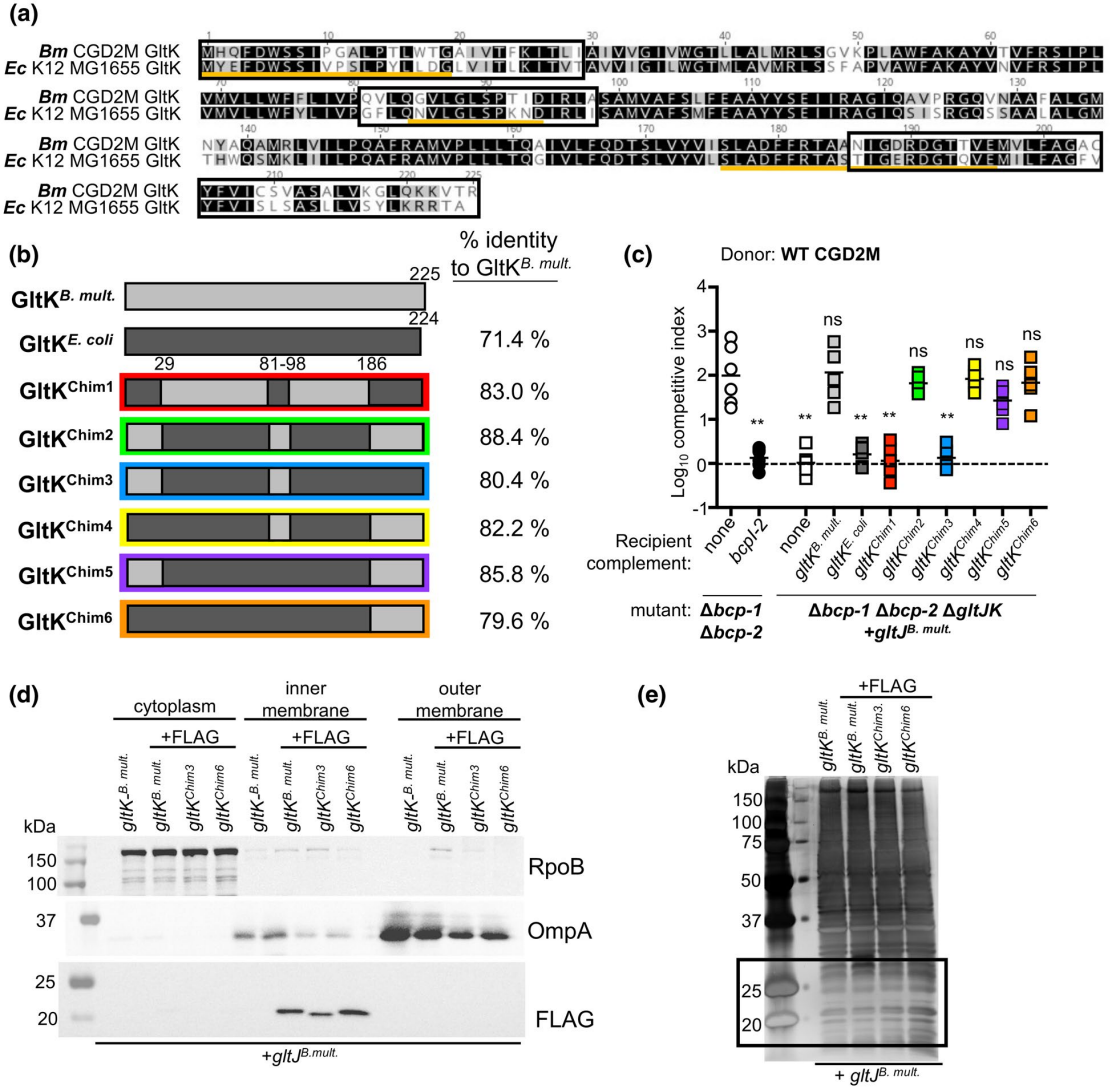
Contribution of GltK to the specificity of the BcpA2 translocation. (a) Amino acid alignment of GltK from *Burkholderia multivorans* CGD2M and *Escherichia coli* MG1655. Yellow lines indicate the predicted periplasmic regions and black boxes show the regions changed in the constructed chimeras. (b) Graphic representation of the GltK proteins from *B. multivorans* and *E. coli* and the chimeras constructed with indicated residue numbers. Percent identity of each protein/chimera to *B. multivorans* CGD2M GltK is shown to the right. (c) Wild‐type donor bacteria were competed against Δ*bcp‐1* Δ*bcp‐2* recipient cells (open circles), Δ*bcp‐1* Δ*bcp‐2* recipients carrying *bcpI‐2* at an *att*Tn7 site (black circles), and Δ*bcp‐1* Δ*bcp‐2* Δ*gltJK* recipient cells complemented at each of two *att*Tn7 sites with empty vectors (none, white squares) or P_S12_‐driven *B. multivorans gltJ* and the indicated *gltK* allele: *B*. *multivorans gltK* (gray squares), *E. coli gltK* (dark gray squares), or genes to produce chimeric GltK proteins *gltK^Chim1^* (red squares), *gltK^Chim2^* (blue squares) *gltK^Chim3^* (green squares), *gltK^Chim4^* (orange squares), *gltK^Chim5^* (purple squares), or *gltK^Chim6^* (yellow squares). Competitive indices (CI) for bacteria sampled from the edge of a colony biofilm after 24 hr co‐culture on agar are shown. CI was calculated as (output donor CFU/recipient CFU) divided by (input donor CFU/recipient CFU). Symbols represent CI from one biological replicate and bars show the mean (*n* = 6 for two independent experiments). Dashed line shows CI = 1 (no competition). Statistical differences were determined with two‐tailed Student’s *t*‐test. **p < .01; compared to corresponding recipient cells with no *bcpI*. (d) Western blots of subcellular fractions of Δ*bcp‐1* Δ*bcp‐2* Δ*gltJK* bacteria complemented at each of two *att*Tn7 sites with P_S12_‐driven *B. multivorans gltJ* and the indicated *gltK* allele: *B*. *multivorans gltK* (untagged), or genes to produce chimeric GltK‐FLAG proteins *gltK^Chim3^*‐*FLAG* and *gltK^Chim6^*‐*FLAG*. Equal amounts of each fraction (cytoplasmic, outer membrane [Sarkosyl‐soluble], and inner membrane [Sarkosyl‐insoluble]) were resolved on 12% sodium dodecyl sulfate‐polyacrylamide gel electrophoresis (SDS‐PAGE) gels and blots probed with anti‐ FLAG peptide (bottom), anti‐*E. coli* RNA Polymerase β subunit (RpoB; top), and anti‐*Salmonella typhii* Outer Membrane protein A (OmpA, middle) antibodies. Expected masses for GltK, OmpA and RpoB are ~26, ~35 and 150, respectively. (e) Equal amounts of the inner membrane fraction samples shown in d) (derived from Δ*bcp‐1* Δ*bcp‐2* Δ*gltJK* mutant bacteria carrying the indicated *gltJ* and *gltK* alleles) were resolved on 12% SDS‐PAGE and visualized by silver staining. Boxed region indicates region shown in in FLAG Western Blot in d)

We hypothesized that the functional difference between GltJK*^B. multi^* and GltJK*^E. coli^* for *B. multivorans* CDI could be used as a tool to define the region(s) of the transporters required for BcpA import. We predicted that interaction of incoming BcpA‐2‐CT polypeptides would occur with the periplasmic region(s) of the GltJK channel. Prediction of transmembrane helices and protein topology indicated that several regions containing sequence divergence between GltK*^B. multi^* and GltK*^E. coli^* and, to a lesser extent between the GltJ proteins, occurred within predicted periplasmic regions (Figures 6a and 7a). To test this hypothesis and map the region(s) required for *translocation* specificity, we constructed a series of chimeric GltJ and GltK proteins that contained heterologous sequence within one or several predicted periplasmic regions (Figures 6b and 7b).

**FIGURE 8:**
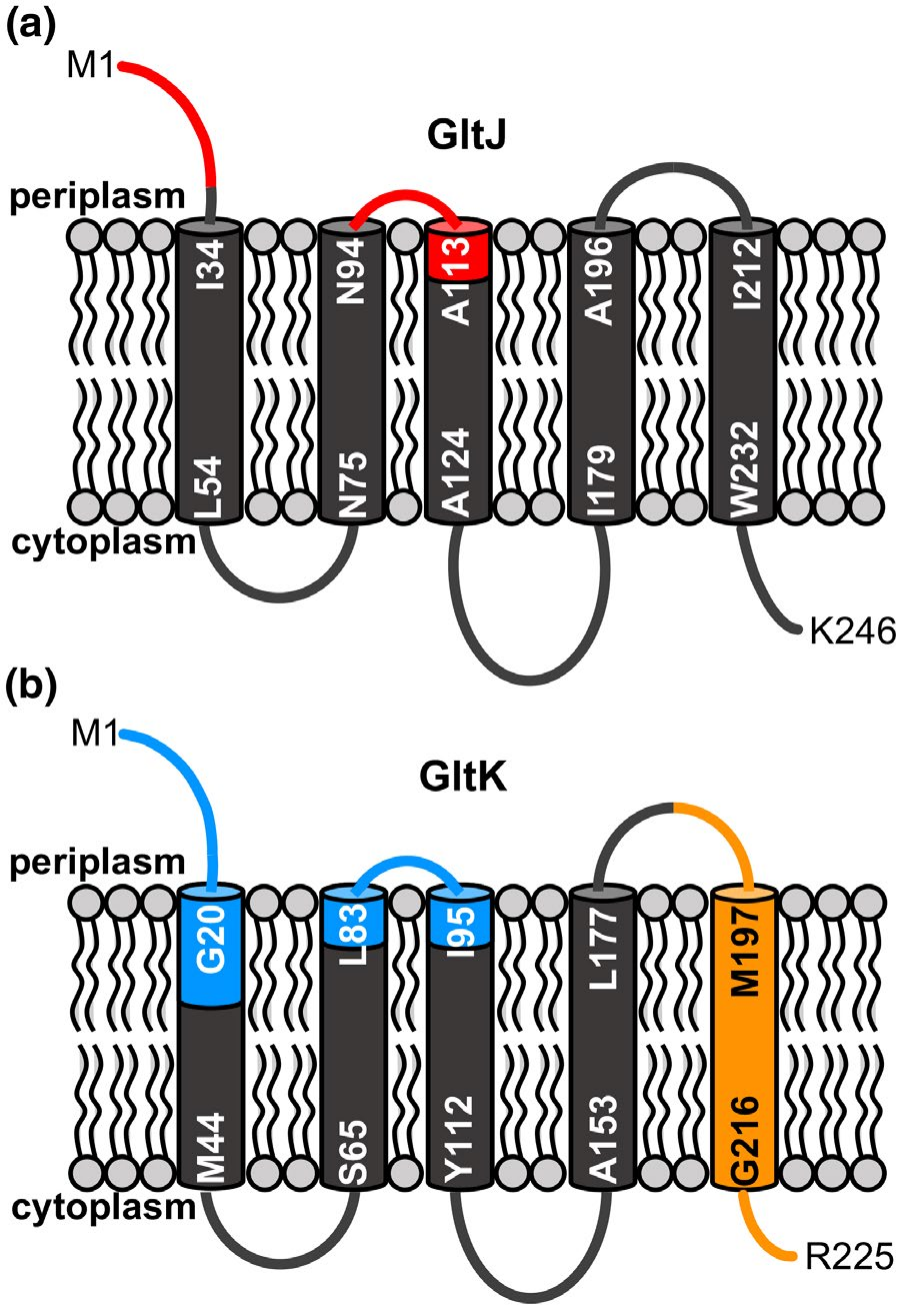
Schematic of predicted GltJ and GltK membrane topologies and contribution to BcpA‐CT‐2 translocation specificity. (a) Predicted GltJ membrane topology, showing the two putative periplasmic region(s) altered in the chimeric GltJ proteins (red). (b) Predicted GltK membrane topology, highlighting the three region(s) altered in chimeric GltK proteins. Blue shading denotes regions that did not contribute significantly to BcpA‐CT‐2 translocation specificity and orange shading shows the region that required *Burkholderia* sequence for BcpA‐CT‐2 translocation. Residue numbers for predicted transmembrane helices are indicated

Chimeric *gltJ* genes were used to complement Δ*bcp‐1*Δ*bcp‐2* Δ*gltJK* mutant recipient bacteria that expressed *B. multivorans gltK*. Replacement of two predicted periplasmic regions (residues 1–19 and 95–116) of GltJ*^B. multi^* with *E. coli* sequences decreased the ability of the resulting chimeric protein, GltJ^Chim1^, to confer CDI susceptibility to recipient cells (Figure 6c), suggesting that these regions are important for the ability of GltJ to mediate BcpA‐2 translocation. However, the chimeric GltJ^Chim1^ retained significantly more function than GltJ*^E. coli^*, indicating that other sequences within *B. multivorans* GltJ are also required for BcpA‐2 transport. Similarly, replacement of the predicted periplasmic N‐terminus of GltJ*^E. coli^* (residues 1–24) with *B. multivorans* sequence resulted in a chimeric protein, GltJ^Chim2^, that increased CDI susceptibility as compared to recipient cells producing GltJ*^E. coli^*, although not to the same level as the complete *B. multivorans* GltJ. Together, these data suggest that several *B. multivorans*‐specific putative periplasmic regions of GltJ (Figure 8a) contribute to entry of BcpA‐2 into recipient cells, but other regions of the protein are also necessary for this function.

### 2.9 C‐terminus of *B. multivorans* GltK is sufficient for BcpA‐2 translocation specificity

To determine the contribution of GltK regions to the species‐specificity of BcpA‐2 translocation, chimeric GltK proteins (Figure 7b) were produced in Δ*bcp‐1*Δ*bcp‐2* Δ*gltJK* mutant recipient bacteria that expressed *B. multivorans gltJ* (Figure 7c). Unlike *E. coli* GltJ, which was partially functional for BcpA‐2 translocation, *E. coli* GltK did not confer any CDI susceptibility to recipient cells (Figures 5b and 7c). Similarly, replacement of three regions of *B. multivorans* GltK that contain predicted periplasmic domains (residues 1–29, 81–98, and 186–225) with *E. coli* sequences rendered the resulting chimeric protein, GltK^Chim1^, nonfunctional for BcpA‐2 translocation (Figure 7c). By contrast, swapping these same regions of *E. coli* GltK with the corresponding *B. multivorans* sequences, resulting in the chimeric protein GltK^Chim2^, restored the ability of this protein to mediate CDI in recipient cells.

Based on this “repaired” GltK^Chim2^ protein, we constructed subsequent chimeras in the *E. coli* GltK backbone that contained two of the three *B. multivorans* sequence regions to test the contribution of each region to BcpA‐2 translocation. Of these, GltK^Chim3^, containing the N‐terminal and center *B. multivorans* regions (Figure 8b), was nonfunctional in recipient cells. GltK^Chim4^, containing the center and C‐terminal regions, and GltK^Chim5^, containing the N‐ and C‐terminal regions, both enabled CDI in recipient cells to levels that did not differ from the CDI susceptibility of bacteria producing full‐length *B. multivorans* GltK. These results indicate that chimeric GltK proteins containing *B. multivorans* sequence at their C‐termini (GltK^Chim2^, GltK^Chim4^, and GltK^Chim5^) were able to mediate BcpA‐2 translocation, while proteins containing *E. coli* sequence within this region (GltK^Chim1^ and GltK^Chim3^) could not.

To test the hypothesis that the GltK C‐terminus mediates the specificity of BcpA‐2 entry, we expressed *gltK^Chim6^* in recipient cells, producing a protein composed of the *E. coli* N‐terminus (residues 1–185) and *B. multivorans* C‐terminus (residues 186–225; Figures 7b and 8b). Recipient cells producing GltK^Chim6^ were strongly outcompeted by *B. multivorans* donor cells (Figure 7c), indicating that the chimeric protein was functional for BcpA‐2 translocation and suggesting that sequences in the GltK C‐terminus are responsible for the specificity of BcpA‐2 for *Burkholderia* GltK.

To determine whether the functional differences among the chimeric GltK proteins were due to defects in abundance or localization, we generated FLAG‐tagged versions of one functional (GltK^Chim6^) and one nonfunctional (GltK^Chim3^) chimera. Similar to the full‐length *B. multivorans* and *E. coli* GltK‐FLAG proteins, GltK^Chim3^‐FLAG and GltK^Chim6^‐FLAG were detected predominantly in the inner membrane fractions (Figure 7d,e). Although we cannot rule out the possibility that chimeric GltK are improperly folded in the *B. multivorans* membrane, these data suggest that GltK abundance or localization do not account for the differences in CDI susceptibility mediated by GltK^Chim3^ and GltK^Chim6^.

The *B. multivorans* and *E. coli* GltK proteins share 56% identity within the C‐terminal region included in GltK^Chim6^. Alignment of additional GltK proteins shows that this C‐terminal region is highly conserved among *Burkholderia* species, diverging slightly in the related species *Ralstonia solanacearum* and more significantly in *E. coli* and other γ‐proteobacteria (Figure S6). Together, these results support the model that *Burkholderia*‐specific sequences within the GltK C‐terminus (Figure 8b) are needed for translocation of BcpA‐2‐CT to the recipient cell cytoplasm.

## 3 DISCUSSION

In this study, we demonstrated that inner membrane proteins GltJK are required for entry of CDI system protein BcpA‐2 into *B. multivorans* recipient cells. Constituting the permease component of a predicted ABC‐type transporter, both GltJ and GltK were required for BcpA‐2 import. Although *E. coli* GltJ and GltK share significant sequence identity with their *B. multivorans* homologs, expression of *E. coli gltJK* did not confer BcpA‐2 susceptibility to recipient cells. These results suggest that BcpA‐2 requires *Burkholderia*‐specific sequences within GltJK for translocation and provided a useful tool to begin probing the mechanism of BcpA cytoplasmic entry. Using chimeric proteins, the specificity determinant was mapped to the C‐terminus of GltK. The presence of *B. multivorans* sequences at the GltK C‐terminus was both necessary and sufficient to render recipient cells sensitive to BcpA‐2 toxicity during interbacterial competition, suggesting that BcpA‐2‐CT may interact with this region.

Studies examining the structure and function of ABC‐type transporters offer clues toward possible roles for the GltK C‐terminal region in BcpA‐2 import. Topology models (UniProt) predict that this region contains a periplasmic region (residues 176–196), a transmembrane helix (residues 197–217), and a cytoplasmic region (residues 218–224; UniProt Consortium, 2019). Consistent with these models, studies using *E. coli* GltK‐PhoA and ‐GFP fusions demonstrated that the GltK C‐terminus is located in the cytoplasm (Daley et al., 2005). This region is also distinct from sequences shown in other ABC transporters to mediate interactions with the ATPase subunits (Rees et al., 2009).

We predicted that periplasmic regions of GltK, available to interact with periplasmic BcpA‐2‐CT that is received from neighboring bacteria, would be responsible for the specificity of BcpA‐2 for GltK*^B. multi^* (Figures 8b and S6). However, only two amino acids within the C‐terminal predicted periplasmic region vary between the *B. multivorans* and *E. coli* proteins, N186 and T193 (T186 and Q193 in GltK*^E. coli^*). Of these, it is unlikely that N186 contributes specificity because *P. luminescens* TTO1 GltK, which is expected to be the native receptor for CdiA‐CT^TTO1^, shares the *B. multivorans* sequence (N186). A greater number of amino acid differences are found within the region’s predicted transmembrane helix (7 substitutions) and the cytoplasmic C‐terminus (4 substitutions). It is not known how BcpA‐CT or CdiA‐CT polypeptides translocate across the recipient cell cytoplasmic membrane nor the precise role that the identified inner membrane receptors, like GltJK, play in this process. Perhaps interactions of BcpA‐CT with membrane‐embedded or channel‐exposed portions of the GltK transmembrane helix or cytoplasmic region are necessary for translocation. Consistent with this hypothesis, a transmembrane helix of SecY was recently found to contribute to cytoplasmic transport of an *E. coli* CdiA‐CT (Jones et al., 2021).

Both GltJ and GltK were required for BcpA‐2 intoxication, although it is unknown whether both proteins participate directly in BcpA‐CT translocation. Our results also indicate that BcpA‐2 is only somewhat specific for *Burkholderia* GltJ, as recipient cells expressing *E. coli gltJ* were partially susceptible to CDI. Inner membrane levels of GltK did not depend on the presence of GltJ, suggesting that GltJ is not necessary for GltK localization or abundance. Chimeric GltJ proteins indicated that several periplasmic regions have minor contributions to the limited specificity of BcpA‐CT‐2 for GltJ*^B. multi^*. GltJ protein levels were also not examined here, so it is possible that defects in the localization or stability of heterologous GltJ contributed to our results. The precise function of GltJ in BcpA‐CT translocation remains unclear, but these results suggest that it plays a supporting role. Future examination of GltJ, particularly focusing on non‐periplasmic regions, may clarify its contribution to BcpA translocation.

In an attempt to swap GltJK specificity, our results demonstrating the inability of a chimeric BcpA^CGD2M^‐CdiA^TTO1^ protein to mediate CDI were somewhat expected. Unlike *E. coli*‐type CdiA proteins, for which functional chimeric proteins can be constructed by grafting the CdiA‐CT from other bacterial species (including CdiA‐CT^TTO1^) onto an *E. coli* CdiA N‐terminus (Aoki et al., 2010; Willett et al., 2015), *Burkholderia* BcpA proteins do not appear as modular. BcpA proteins fall into several subclasses and functional chimeras can be constructed by swapping BcpA‐CT domains among subclass‐1 or among subclass‐2 proteins (Anderson et al., 2014; Nikolakakis et al., 2012), but not between the subclasses (Anderson et al., 2014). Thus, CT domains of even closely‐related *Burkholderia* BcpA proteins cannot always be swapped to generate functional chimeras, due to yet unknown mechanisms. While it is possible that chimeric BcpA^CGD2M^‐CdiA^TTO1^ did not mediate CDI due to improper conformation of *E. coli* GltJK when produced in *B. multivorans* or the lack of a possible cytoplasmic “activator” protein (Diner et al., 2012; Koskiniemi et al., 2014), these previous data support the hypothesis that chimeric BcpA^CGD2M^‐CdiA^TTO1^, which fused a distantly‐related CT domain onto a BcpA N‐terminus, was nonfunctional. *B. multivorans* BcpA‐2 is an atypical BcpA, appearing to have a CdiA‐like “VENN” motif rather than the “Nx(E/Q)LYN” that precedes many BcpA‐CT domains (Myers‐Morales et al., 2019). Despite this commonality with CdiA, the results here suggest that BcpA‐2 is functionally similar to typical BcpA proteins in its lack of modularity. These results highlight gaps in the understanding of the molecular requirements for proper *Burkholderia* BcpA secretion, processing, and translocation into recipient cells.

The regulatory mechanisms controlling expression of *B. multivorans gltIJKL* have not been delineated. In *Salmonella*, expression of *gltIJKL* and other amino acid transporter‐encoding genes is regulated by nutrient availability and small regulatory RNAs (Miyakoshi et al., 2015; Sharma et al., 2007). Whether modulation of cellular concentrations of GltJK (or other BcpA/CdiA receptors) by environmental conditions could impact CDI susceptibility remains an interesting outstanding question.

Mechanisms of CDI systems found in *Burkholderia* species are distinct and the receptors that mediate BcpA entry into recipient cells are largely unknown. This study identified proteins likely composing the inner membrane receptor for one *Burkholderia* BcpA‐CT variant. A distinct inner membrane protein has also been identified as a probable receptor for a *Burkholderia pseudomallei* BcpA‐CT (Koskiniemi et al., 2015; Willett et al., 2015). Evidence from *B. thailandensis* also indicates that bacteria producing altered LPS are resistant to a specific BcpA variant, although it is unclear whether LPS functions as the direct outer membrane receptor or whether altered LPS indirectly affects BcpA entry (Koskiniemi et al., 2015).

Our findings support the model that BcpA and CdiA proteins are exchanged between closely related species. This exchange is likely limited predominantly by interactions between BcpA/CdiA and their cognate outer membrane receptors, although it is possible that inner membrane receptors like GltJK also contribute. It is unclear whether the specificity determinant identified here, the GltK C‐terminus, is also important for translocation of *E. coli* GltJK‐utilizing CdiA‐CT toxins. Similar specificity may exist for other CDI system effectors, but the extent to which inner membrane receptor variability restricts natural exchange of CdiA/BcpA proteins remains to be determined. The recent finding that some CdiA proteins can be exchanged cross‐species among *Enterobacteriaceae* spp. implies that any inner membrane receptor variation among these species does not substantially limit CdiA‐CT delivery (Virtanen et al., 2019). We expect that during natural CDI system activity, BcpA‐2‐CT^CGD2M^ mainly encounters *Burkholderia* GltJK, rather than γ‐proteobacterial GltJK. Thus, it is not unexpected that the translocation domain of BcpA‐2‐CT^CGD2M^ evolved to recognize the GltJK homolog found in its native recipient cells. Overall, the results here contribute to the model of CDI system protein exchange and provide insight into the mechanism of BcpA translocation across the cytoplasmic membrane.

## 4 EXPERIMENTAL PROCEDURES

### 4.1 Bacterial strains and culture conditions

Bacterial strains used in this study are listed in Table 1. *B. multivorans* CGD2M was isolated from a patient with chronic granulomatous disease. *B. thailandensis* E264 is an environmental isolate (Brett et al., 1998). Plasmids were maintained in *E. coli* DH5α and mated into *B. multivorans* using the donor *E. coli* strain RHO3 (López et al., 2009). *B. multivorans* was cultured in low salt Luria‐Bertani medium (LSLB, 0.5% NaCl), supplemented, as appropriate, with 20 µg/ml chloramphenicol, 250 µg/ml kanamycin, 25–50 µg/ml tetracycline, or 0.2% glucose. Overnight cultures were aerated for ∼18 hr at 37℃ to OD_600_ ∼2, centrifuged at 16,000×*g* for 2 min, washed, and resuspended in fresh LSLB broth or sterile phosphate buffered saline (PBS) for subsequent use. *E. coli* strains were cultured in LSLB supplemented, as appropriate, with 100 µg/ml ampicillin, 50 µg/ml kanamycin, 10 µg/ml tetracycline, 200 µg/ml 2,6‐diaminopimelic acid (for RHO3 strains), or 0.2% glucose.

**TABLE 1:** Bacterial strains and mutants used in this study

Strain	Description	References
*E. coli* RHO3	Conjugation donor strain, Δ*asd*	López et al. (2009)
*E. coli* DH5α	*E. coli* cloning strain	
CGD2M	Wild‐type *Burkholderia multivorans* isolated from patient with chronic granulomatous disease	
Δ*bcp‐2*	In‐frame deletion of *bcpAIB‐2* in CGD2M	Myers‐Morales et al. (2019)
Δ*bcp‐1* Δ*bcp‐2*	In‐frame deletion of *bcpAIOB‐1* and *bcpAIB‐2* in CGD2M	Myers‐Morales et al. (2019)
Δ*gltJK*	In‐frame deletion of *gltJ* and *gltK* in CGD2M	This study
Δ*bcp‐1* Δ*bcp‐2* Δ*gltJK*	In‐frame deletion of *gltJ* and *gltK* in CGD2M Δ*bcp‐1* Δ*bcp‐2* mutant	This study
Δ*bcp‐2* Δ*gltJK*	In‐frame deletion of *gltJ* and *gltK* in CGD2M Δ*bcp‐2* mutant	This study
Δ*bcp‐1* Δ*bcp‐2* Δ*gltI*	In‐frame deletion of *gltI* in CGD2M Δ*bcp‐1* Δ*bcp‐2* mutant	This study
CGD2M‐30	CGD2M with the native *bcpA‐2‐CT* and *bcpI‐2* replaced with *Burholderia cenocepacia* Bp8968 *bcpA*‐CT, *bcpI* and DF018_16545, starting at the VENN of *bcpA‐2*	This study
CGD2M‐32	CGD2M with the native *bcpA‐2‐CT* and *bcpI‐2* replaced with *Photorhabdus luminecens* TTO1 *cdiA*‐CT and *cdiI*, starting at the VENN of *bcpA‐2*	This study

### 4.2 Genetic manipulations

Plasmids used in this study are listed in Table 2. All plasmids were confirmed by DNA sequencing (Eurofins Genomics or ACGT, Inc.) and bacterial mutant strains verified by PCR.

**TABLE 2:** Plasmids used in this study

Strain or plasmid	Description	References
pEXKm5	*sacB*‐based allelic exchange vector, Kan^R^	López et al. (2009)
pUC18T‐miniTn7‐kan	Plasmid for *att*Tn7 site delivery, Amp^R^, Kan^R^	Choi et al. (2005)
pTNS3	Helper plasmid for *att*Tn7 site delivery, Amp^R^	Choi et al. (2008)
pUCS12	P_S12_ promoter in pUC18T‐miniTn7‐kan, Amp^R^, Kan^R^	Anderson et al. (2012)
pUCCm	Cm^R^ plasmid for *att*Tn7 site delivery, Amp^R^, Cm^R^	Anderson et al. (2012)
pUCTet	Tet^R^ plasmid for *att*Tn7 site delivery, Amp^R^, Tet^R^	Anderson et al. (2012)
pECG69	*bcpI‐1* driven by P_S12_ in pUCTet, Amp^R^, Tet^R^	Myers‐Morales et al. (2019)
pECG70	*bcpI‐2* driven by P_S12_ in pUCTet, Amp^R^, Tet^R^	Myers‐Morales et al. (2019)
pECG74	First ~500 bp of *bcpA‐1* cloned 3′ to P_S12_ in pUCS12, for constitutive *bcpAIOB‐1,* Kan^R^	Myers‐Morales et al. (2019)
pUT‐miniTn5‐Kn	Used for transposon mutagenesis, Kan^R^	de Lorenzo et al. (1990)
pScrhaB3	Used for expression of genes under rhamnose induction, Kan^R^	Cardona and Valvano (2005)
pTMM009	Deletion allele for *gltJ* and *gltK* in pEXKm5, Kan^R^	This study
pTMM012	*gltJK^B. mult^*. operon (driven by P_S12_) in pUC18T‐miniTn7‐kan, Amp^R^, Kan^R^	This study
pTMM013	*gltJK^E. coli^* operon (driven by P_S12_) in pUC18T‐miniTn7‐kan, Amp^R^, Kan^R^	This study
pTMM016	*gltJK^B. thai^* operon (driven by P_S12_) in pUC18T‐miniTn7‐kan, Amp^R^, Kan^R^	This study
pTMM018	*gltJ^E. coli^* (driven by P_S12_) in pUC18T‐miniTn7‐kan, Amp^R^, Kan^R^	This study
pTMM019	*gltK^E. coli^* (driven by P_S12_) in pUCTet, Amp^R^, Tet^R^	This study
pTMM020	*gltJ^B. mult^* (driven by P_S12_) in pUC18T‐miniTn7‐kan, Amp^R^, Kan^R^	This study
pTMM021	*gltK^B. mult^*. (driven by P_S12_) in pUCTet, Amp^R^, Tet^R^	This study
pJChim1	GeneArt plasmid containing *gltJ* ^Chim1^ in pMK‐RQ backbone, Kan^R^	This study
pKChim1	GeneArt plasmid containin *gltK* ^Chim1^ in pMK‐RQ backbone, Kan^R^	This study
pKChim2	GeneArt plasmid containin *gltK* ^Chim2^ in pMA‐RQ backbone, Amp^R^	This study
pTiT01	GeneArt plasmid for *bcpA‐2* chimera, includes ~500 bp upstream of CGD2M *bcpA‐2‐CT* in‐frame with *cdiA‐CT* and *cdiI* from *Photorhabdus luminescens* TTO1, and ~500 bp downstream of CGD2M *bcpI‐2* in pMK‐RQ backbone, Kan^R^	This study
pBp8968	GeneArt plasmid for *bcpA‐2* chimera, includes ~500bp upstream of CGD2M *bcpA‐2‐CT* in‐frame with *bcpA‐CT, bcpI* and DF018_16545 from *Burkholderia cenocepacia* Bp8968, and ~500 bp downstream of CGD2M *bcpI‐2* in pMK‐RQ backbone, Kan^R^	This study
pTMM022	*gltJ* ^Chim1^ (driven by P_S12_) in pUC18T‐miniTn7‐kan, Amp^R^, Kan^R^	This study
ipTMM023	*gltK* ^Chim1^ (driven by P_S12_) in pUCTet, Amp^R^, Tet^R^	This study
pTMM024	*gltIJKL^E. coli^* complete locus (driven by P_S12_) in pUC18T‐miniTn7‐kan, Amp^R^, Kan^R^	This study
pTMM025	CGD2M *bcpA‐2*‐*CT* in pSCrhaB3, Kan^R^	This study
pTMM027	*gltK* ^Chim2^ (driven by P_S12_) in pUCTet, Amp^R^, Tet^R^	This study
pTMM030	Allelic exchange plasmid for *bcpA‐2* chimera with *bcpA‐CT*, *bcpI* and DF018_16545 from *Burkholderia cenocepacia* Bp8968 and ~500 bp flanking CGD2M sequence in pEXKm5, Kan^R^	This study
pTMM031	*B. cenocepacia* Bp8968 *bcpI* (driven by P_S12_) in pUC18T‐miniTn7‐kan, Amp^R^, Kan^R^	This study
pTMM032	Allelic exchange plasmid for *bcpA‐2* chimera with *cdiA‐CT* and *cdiI* from *Photorhabdus luminescens* TTO1 and ~500 bp flanking CGD2M sequence in pEXKm5, Kan^R^	This study
pTMM034	Deletion allele for *gltI* in pEXKm5, Kan^R^	This study
pTMM035	*gltI* from CGD2M (driven by P_S12_) in pUC18T‐miniTn7‐kan, Amp^R^, Kan^R^	This study
pTMM036	*gltJ* ^Chim2^ (driven by P_S12_) in pUC18T‐miniTn7‐kan, Amp^R^, Kan^R^	This study
pTMM037	*gltK* ^Chim3^ (driven by P_S12_) in pUCTet, Amp^R^, Tet^R^	This study
pTMM038	*gltK* ^Chim4^ (driven by P_S12_) in pUCTet, Amp^R^, Tet^R^	This study
pTMM039	*gltK* ^Chim5^ (driven by P_S12_) in pUCTet, Amp^R^, Tet^R^	This study
pTMM040	*gltK* ^Chim6^ (driven by P_S12_) in pUCTet, Amp^R^, Tet^R^	This study
pTMM041	*gltK^B. mult^* ‐FLAG (driven by P_S12_) in pUC18T‐miniTn7‐kan, Amp^R^, Tet^R^	This study
pTMM042	*gltK^E. coli^* ‐FLAG (driven by P_S12_) in pUCTet, Amp^R^, Tet^R^	This study
pTMM046	*gltK* ^Chim6^‐FLAG (driven by P_S12_) in pUCTet, Amp^R^, Tet^R^	This study
pTMM047	*gltK* ^Chim3^‐FLAG (driven by P_S12_) in pUCTet, Amp^R^, Tet^R^	This study

Abbreviations: Amp, ampicillin; Cm, chloramphenicol; Kan, kanamycin; Tet, tetracycline.

In‐frame deletion mutations of *gltJK* and *gltI* were constructed by allelic exchange. DNA fragments corresponding to ~500 bp 5′ to *gltJ* (BURMUCGD2M_3194, including the first three codons of *gltJ*) and ~500 bp 3′ to *gltK* (BURMUCGD2M_3193, including the last ten codons of *gltK*), were joined by overlap PCR and cloned into EcoRI‐digested allelic exchange vector pEXKm5, resulting in plasmid pTMM009. A fragment consisting of ~500 bp 5′ to *gltI* (BURMUCGD2M_3196, including the first ten codons of *gltI*) and ~500 bp 3′ to *gltI* (including the last 9 codons of *gltI*) was generated and cloned into pEXKm5, resulting in plasmid pTMM034. Both plasmids were used for allelic exchange in CGD2M as described (López et al., 2009).

DNA fragments containing *bcpA‐2* chimeras with *Photorhabdus luminescens* TTO1 (TTO1) *cdiA*‐CT or *B. cenocepacia* Bp8968 (Bp8968) *bcpA*‐CT were ordered from Thermo Fisher Scientific (GeneArts Strings Gene Synthesis) flanked by EcoRI sites on each end. The regions ordered contained 530bp upstream of the CGD2M *bcpA‐2* (BURMUCGD2M_2430) VENN ending in nucleotide 9,297 from *bcpA2*, followed by either the TTO1 *cdiA*‐CT (plu0548) starting at 12,727bp of CdiA (TTO1 VENN) and ending at the stop codon of TTO1 *cdiI* (plu0547) or the Bp8968 *bcpA*‐CT (DF018_16555) starting at 9,297bp of *B. cenocepacia* Bp8968 BcpA (Bp8968 VENN) and ending at the stop codon of DF018_16545 (extra open reading frame after *B. cenocepacia* Bp8969 *bcpI*). Either region was followed by 569bp downstream of the CGD2M *bcpI‐2* (BURMUCGD2M_2429) stop codon. Both chimeric fragments were received in plasmids we designated pTTO1 and pBp8968, respectively. After digest of plasmids with EcoRI, the region containing the chimeric inserts was band purified and ligated into EcoRI digested pEXKm5, resulting in plasmids pTMM032 (TTO1 chimera) and pTMM030 (Bp8968 chimera) which were used for allelic exchange in CGD2M as described (López et al., 2009) resulting in strains with chimeric *bcpA‐2* alleles designated CGD2M‐32 and CGD2M‐30 respectively. For competitions, a gene conferring chloramphenicol resistance was added to CGD2M‐32 with plasmid pUCCm (previously described (Anderson et al., 2012)) and a gene encoding kanamycin resistance was added to CGD2M‐30 with plasmid pUC18T‐miniTn7T‐Kan by delivery to one of the *att*Tn7 sites (Choi et al., 2008).

Constitutive expression of the *bcp‐1* locus in CGD2M, achieved by integration of the P_S12_ promoter (promoter of *B. thailandensis rpsL*, encoding the ribosomal S12 subunit) immediately 5′ to *bcpA‐1* with plasmid pECG74, has been described previously (Myers‐Morales et al., 2019).

Deletion mutants were complemented by introducing chromosomal copies of the indicated genes to *att*Tn7 sites using miniTn7 transposition as described previously (Choi et al., 2008). *gltJK* was PCR amplified from CGD2M, *Bukholderia thailandensis* E264, or *E. coli* DH5α (DH5α) and cloned 3′ to P_S12_ in pUCS12 (Anderson et al., 2012), resulting in pTMM012, pTMM016 and pTMM013, respectively. The complete *E. coli gltIJKL* locus was PCR‐amplified from DH5α and cloned 3′ to P_S12_ in pUCS12 resulting in pTMM024. *gltJ* was PCR‐amplified from CGD2M or DH5α and cloned 3′ to P_S12_ in pUCS12 resulting in pTMM020 and pTMM018, respectively. *gltK* was PCR‐amplified from CGD2M or DH5α and cloned 3′ to P_S12_ in pUCTet (Anderson et al., 2012), resulting in pTMM021 and pTMM019, respectively. *gltI* was PCR amplified from CGD2M and cloned 3′ to P_S12_ in pUCS12 resulting in pTMM035. *B. cenocepacia* Bp8968 *bcpI* was amplified from pBp8968 and cloned 3′ to P_S12_ in pUCS12, resulting in plasmid pTMM031. Plasmids pECG69 and pECG70, encoding a P_S12_‐driven copy of CGD2M *bcpI‐1* and *bcpI‐2,* respectively, have been described previously (Myers‐Morales et al., 2019). For use in competition assays, the mutants were tagged with a tetracycline resistance cassette at a second *att*Tn7 site using pUCTet or a kanamycin resistance cassette using pUC18T‐miniTn7T‐Kan (Myers‐Morales et al., 2019). The mutant expressing *gltJ* alone (Kan^R^) was additionally marked with a tetracycline resistance cassette at a second *att*Tn7 site using pUCTet. Similarly, the mutant expressing *bcpI‐2* (Tet^R^) was additionally tagged with a kanamycin resistance cassette using pUC18T‐miniTn7T‐Kan.

DNA fragments containing chimeric *gltJ* and *gltK* genes composed of CGD2M and *E. coli* sequences were ordered from Thermo Fisher Scientific (GeneArts Strings Gene Synthesis). Chimeric gene *gltJ^Chim1^* consists of CGD2M *gltJ*. coding sequence with nucleotides 7–57 and 281–345 replaced with nucleotides 7–54 and 281–345 from *E. coli* MG1655 *gltJ* (b0654), respectively. Flanked by 5′ HindIII and 3′ StuI sites, restriction digestion was used to clone *gltJ^Chim1^* into pUCS12 downstream of P_S12_, resulting in plasmid pTMM022. Chimeric gene *gltK* ^Chim1^ consists of CGD2M *gltK* coding sequence with nucleotides 4–87, 241–294, and 553–678 replaced with nucleotides 4–87, 241–294, and 553–675 from *E. coli* MG1655 *gltK* (b0653), respectively. Chimeric gene *gltK* ^Chim2^ is the opposite of *gltK* ^Chim1^, consisting of *E. coli* MG1655 *gltK* coding sequence with nucleotides 4–87, 241–294, and 553–678 replaced with nucleotides 4–87, 241–294, and 553–678 from CGD2M *gltK*. Flanked by 5′ HindIII and 3′ KpnI restriction sites, restriction digestion was used to clone *gltK* ^Chim1^ and *gltK* ^Chim2^ genes 3′ to P_S12_ in pUCTet, resulting in pTMM023 and pTMM027, respectively.

Chimeric gene *gltJ* ^Chim2^ consists of *E. coli* MG1655 *gltJ* coding sequence with nucleotides 7–75 replaced with nucleotides 7–78 from CGD2M *gltJ*. *gltJ* ^Chim2^ was made by amplifying P_S12_ and the 5′ end of *gltJ^B. mult^*. (nucleotides 1–78) from pTMM020 and the 3′ end of *gltJ^E. coli^* (nucleotides 76–741) from pTMM018. The two regions were joined by overlap PCR and cloned into pUC18T‐miniTn7T‐Kan, resulting in plasmid pTMM036.

The remaining chimeric *gltK* genes were constructed by restriction digestion of plasmids containing *gltK*, *gltK* ^Chim1^ and *gltK* ^Chim2^, taking advantage of naturally occurring HpaI and BglI restriction sites within the *gltK* sequence. Plasmids pTMM019 (*E. coli gltK*) and pTMM027 (*gltK* ^Chim2^) were digested with SacI and StuI, and the resulting inserts (which include P_S12_ and the *gltK* gene) were further digested with: (1) HpaI to produce 438bp 5′ (H Up) and 519bp 3′ (H down) fragments or (2) BglI to produce 653bp 5′ (B up) and 309bp 3′ (B down) fragments. Combinations of these fragments were cloned into pUCTet: pTMM027 B Up with pTMM019 B Down resulting in pTMM037 (*gltK* ^Chim3^), pTMM019 H Up with pTMM027 H Down resulting in pTMM038 (*gltK* ^Chim4^) and pTMM019 B Up with pTMM027 B Down resulting in pTMM040 (*gltK* ^Chim6^). Plasmid pTMM040 was similarly digested with SacI and StuI and the resulting *gltK* ^Chim6^ insert was subsequently digested with HpaI to produce two fragments, H up (438bp) and H Down (519bp). Fragment H Down from pTMM040 was ligated with pTMM027 H Up into pUCTet to produce pTMM039 (*gltK* ^Chim5^).

Nucleotides encoding a 3x alanine linker and 1x FLAG tag were added to the 3′ end of the indicated *gltK* genes by PCR amplification of *gltK* from CGD2M or DH5α or chimeric *gltK* genes from pTMM040 and pTMM037. The resulting *gltK* genes were cloned 3′ of P_S12_ into pUCTet, resulting in plasmids that produce C‐terminal FLAG‐tagged GltK: pTMM041 (GltK*^B. mult^*‐FLAG), pTMM042 (GltK*^E. coli^*‐FLAG), pTMM046 (GltK^Chim6^‐FLAG), and pTMM047 (GltK^Chim3^‐FLAG).

Nucleotides encoding the C‐terminus of CGD2M BcpA‐2 (nucleotides 9,298–10,272) were amplified with a 5′ primer that added an in‐frame start codon. This fragment was cloned 3′ of P_rham_ in pScrhaB3 (Cardona & Valvano, 2005), resulting in pTMM025.

### 4.3 Transposon mutagenesis selection and arbitrary PCR

Random transposon mutagenesis of CGD2M Δ*bcpAIB‐2* was achieved by delivering pUT‐MiniTn5‐Kn (de Lorenzo et al., 1990) by conjugation as described (López et al., 2009). The mating mixture was serially diluted and plated on LSLB with kanamycin. Colonies were enumerated, collected, and pooled in LSLB with 15% glycerol for storage of the transposon mutant library.

For selection of CDI resistant (CDI^R^) mutants, sequential interbacterial competition assays were used (Anderson et al., 2012; Aoki et al., 2008). Briefly, the transposon pool was cultured in LSLB with kanamycin and mixed at 1:1 ratio with Cm^R^ wild‐type CGD2M. The mixture was serially diluted and plated on antibiotic plates to determine the initial competition ratio. The mixture (20 µl) was spotted on LSLB agar in triplicate, air dried, and incubated at 25℃ for 24 hr. Colony spots were collected with a sterile loop, serially diluted, and plated on antibiotic plates to determine final competition ratios. Kan^R^ transposon mutant colonies were collected from output plates and pooled from all replicates in LSLB with 15% glycerol for storage. This process was repeated three times, using the output pool of transposon mutants as the inoculum for the next round of competition selection.

Transposon insertion sites of CDI^R^ mutants were determined by arbitrary PCR, as described (Intile et al., 2015) with modifications. Genomic DNA was extracted from CDI^R^ mutants and a wild‐type CGD2M control using the Wizard® Genomic DNA Purification system (Promega) according to the manufacturer’s protocol. Nested arbitrary‐primed PCR was performed using this genomic DNA as template and a first round primer annealing to the 3′ end of the transposon (Tn3out, 5′‐CACGCAGATGGGCCGGC) and an arbitrary primer (Arb1, 5′‐GGCCACGCGTCGACTAGTACNNNNNNNNNNACGCC). Products from the first reaction were treated with ExoSAP‐IT™ PCR Product Cleanup Reagent (Applied Biosystems) and used as template in a second, nested PCR reaction using primers Arb2 (5′‐GGCCACGCGTCGACTAGTAC) and Tn3in (5′‐CAAGCGCGAGATGTTCACCGACCC). Second‐round PCR products were analyzed by agarose gel electrophoresis, treated with ExoSAP‐IT™, and the transposon‐chromosome junctions sequenced with primer Tn3seq (5′‐CATCACACGAACAAAGATGG).

### 4.4 Interbacterial competition assay

Interbacterial competition assays were performed as previously described (Anderson et al., 2012), with modifications. Bacteria carrying antibiotic resistance markers were cultured overnight, washed with PBS, and diluted in PBS to OD_600_ = 2. Strains were mixed at a 1∶1 ratio, and 20 µl of the mixture was plated on LSLB agar without antibiotic selection. The culture inoculum was plated on LSLB with antibiotic selection to determine the ratio of each strain at 0 hr. Agar plates were incubated at 25℃ for 24–26 hr. Bacteria were picked from the edge of the resulting colony spots/biofilms with a sterile pipette tip, diluted in PBS, and plated on LSLB with antibiotic selection to quantify each strain at 24 hr. The competitive index (CI) was calculated as the ratio of the inhibitor strain to the target strain at time 24 hr divided by the ratio at time 0 hr. Two to three independent experiments were performed in triplicate.

### 4.5 Intracellular toxicity assay

Plasmids pScrhaB3 and pTMM025 (Rham*‐bcpA‐2*‐CT) were introduced by conjugation into CGD2M Δ*bcpAIOB‐1* Δ*bcpAIB‐2*, CGD2M Δ*bcpAIOB‐1* Δ*bcpAIB‐2* Δ*gltJK* and each strain carrying *bcpI‐2* at an *att*Tn7 site. Conjugation mixtures were incubated on LSLB agar supplemented with DAP at 37℃ for 6 hr. Approximately half of each mating mixture was collected with a sterile polyester swab, streaked for isolation on plates supplemented with kanamycin and 0.2% glucose, incubated ~24 hr at 37℃, and imaged.

### 4.6 Subcellular fractionation and western blotting

Subcellular fractionation of CGD2M was performed by the Sarkosyl extraction method (Filip et al., 1973; Myers‐Morales et al., 2007) with modifications. Briefly, bacterial strains were cultured overnight at 37℃ with shaking in LSLB. Strains were resuspended to an OD_600_ of 4 in HEPES Resuspension Buffer (10 mM HEPES, pH 7.5 supplemented with Roche Complete Mini EDTA‐free Protease Inhibitor Cocktail and Pierce Universal Nuclease for Cell Lysis). Cells were broken by two passages through a chilled French pressure cell (20,000 lb/in^2^), and unbroken cells and large debris were removed by centrifugation at 10,000×*g* at 4℃ for 15 min, followed by an additional centrifugation for 10 min. Total membranes were separated by ultracentrifugation for 15 min at 100,000×*g* at 4℃ and supernatants collected for analysis of the cytoplasmic fraction. Total membranes were washed with HEPES Resuspension Buffer, and the pellet was resuspended in 0.3% (wt/vol) sodium laurylsarcosinate (Sarkosyl) in 10 mM HEPES, pH 7.5 and incubated with rotation at RT for 30 min. Samples were separated by ultracentrifugation at 100,000×*g* at 20℃ and supernatants collected for analysis of Sarkosyl‐soluble (inner membrane‐enriched) proteins. The pellet was washed with 0.3% Sarkosyl Buffer, and the Sarkosyl‐insoluble (outer membrane‐enriched) fraction was resuspended in 10 mM Tris, pH 8 with 1% sodium dodecyl sulfate (SDS) (Tris/SDS buffer). The cytoplasmic and inner membrane fractions were concentrated by methanol−chloroform precipitation, and the resulting pellets were resuspended in Tris/SDS buffer. Protein concentration for all fractions was determined by microplate BCA assay (Pierce).

Equal protein amounts of each fraction were analyzed by sodium dodecyl sulfate‐polyacrylamide gel electrophoresis (SDS‐PAGE) and transferred to nitrocellulose membrane. Immunoblots were probed with rabbit polyclonal anti‐*Salmonella typhii* Outer Membrane protein A (Biomatik) or mouse monoclonals anti‐FLAG M2 (Sigma) and anti‐*E. coli* RNA Polymerase β (Biolegend) and secondary antibodies coupled to IRDye 800CW (Licor). Silver staining of the gels containing the inner membrane (Sarkosyl‐soluble) fractions was used for visualization of protein loading. Immunoblots and gels were imaged on a BioRad ChemiDoc MP Imaging System.

### 4.7 Bioinformatics and statistics

BLASTp analysis using *B. multivorans* BcpA‐2‐CT as a query was used to identify BcpA from *B. cenocepacia* strain Bp8968 (encoded by DF018_16555). Protein sequences were aligned using the ClustalW alignment function in Geneious (v.6.1.8). Transmembrane helices and membrane topology of GltJ and GltK were predicted using Uniprot (UniProt Consortium, 2019) and the TMPred program available via ExPASY‐Swiss Institute of Bioinformatics. The Shapiro‐Wilk normality test was performed on all datasets to determine distribution. Normally distributed data were analyzed by two‐tailed Student’s *t*‐tests. Data failing the normality test were analyzed by the nonparametric Mann−Whitney test. All statistics were performed with GraphPad Prism (v.8).

## CONFLICT OF INTEREST

The authors have no conflict of interest to declare.

## Supporting information

Supplementary Material

Table S1

## Data Availability

The raw data that support the findings of this study are found in the supplementary material (Tables S1 and S2) or available from the corresponding author upon request.
