# Supplementary material for: Burkholderia multivorans requires species‐specific GltJK for entry of a contact‐dependent growth inhibition system protein
Source: Mol Microbiol. 2021 Jul 28;116(3):957–73. doi: 10.1111/mmi.14783 (PMC9291907; doi:10.1111/mmi.14783)
Supplement: Supplementary file 1 — Supplementary Material [file MMI-116-957-s002.pdf]

SUPPLEMENTAL INFORMATION

Burkholderia multivorans requires species-specific GltJK for entry of a contact-dependent growth inhibition system protein

Tanya Myers-Morales, Martha M.S. Sim[1], Tanner J. DuCote[2], and Erin C. Garcia*

Department of Microbiology, Immunology, and Molecular Genetics, University of Kentucky, Lexington, KY 40536

SUPPLEMENTAL INFORMATION

CONTENT LIST

Table S1. Log10 competitive index data for all competition assay figures (separate Excel file, tabulated by Figure).

Table S2. Chromosomal insertion sites of BcpA-2-resistant miniTn5 mutants.

Fig. S1. Alignment of GltJK-utilizing BcpA-CT and CdiA-CT polypeptides.

Fig. S2. Inter-bacterial competition mediated by *B. multivorans* producing chimeric BcpA-CT proteins.

Fig. S3. Detection of putative *B. multivorans* RpoB and OmpA with anti-*E. coli* RpoB and anti-*Salmonella typhii* OmpA antibodies.

Fig. S4. Interbacterial competition with overproduction of GltJK and subcellular localization of *B. multivorans* GltK in the presence and absence of GltJ*.*

Fig. S5. Total inner membrane protein from subcellular fractionation of *B. multivorans* producing heterologous and chimeric GltK-FLAG proteins.

Fig. S6. Alignment of GltK proteins from selected β- and γ-proteobacterial species.

Table S2. Chromosomal insertion sites of BcpA-2-resistant miniTn5 mutants.

Tn mutant[a]	Insertion in gene	Insertion after gene bp #[b]	Identifying sequence[c]
C1*	*gltJ*	168	GTGTATAAGAGTCAGGTGCCNAANAAATGGCTGGCC GCGATCGGCACGGTCTATGTGTCGATCTTCC
C2	*gltJ*	455	TGTATAAGAGTCAGGTGGNANACGTATCGCTACGTG CTGCTGCCGGTCGCCTACCGGATCATCGTGCCGCC
C5	*gltK*	361	TTGTGTATAAGAGTCAGANNNNNNNCCGCGCGGGC AGGTGAACGCG
C6	*gltK*	60	TTGTGTATAAGAGTCAGATCGTCACGTTCAAGATCA CGCTGATCGCGATCGTGGTCGGGATCGTCTGGGGC
C7*	*gltJ*	168	GCACTTGTGTATAAGAGTCAGGTGCCGAACAAATGG CTGGCCGCGATCGGCACGGTCTATGTGTCGATCTTC
C8*	*gltJ*	168	GCACTTGTGTATAAGAGTCAGGTGCCGAACAAATGG CTGGCCGCGATCGGCACGGTCTATGTGTCGATCTTC
C9	*gltK*	586	GTATAAGAGTCAGAGATGGTGCTGTTCGCCGGCGC CTGCTATTTC
C10	*gltK*	482	CGCACTTGTGTATAAGAGTCAGGGCGATCGTGCTGT TCCAGGACACGTCGCTCGTGT
C11†	*gltK*	317	CACTTGTGTATAAGAGTCAGGTTCGAAGCCGCGTAT TATTCCGAGATCATCCG
C14	*gltJ*	359	TTGTGTATAAGAGTCAGGTTCACCGGCGCACGCGTG TGCGAACAGGTGCGC
C15†	*gltK*	317	GTGNATNANAAGNCAGGTTCGAAGCCGCGTATTATT TCCGAAGATCATCCGCGCCGGCATCCAGGCGGTGC
C16*	*gltJ*	168	TTGTGTATAAGAGTCAGGTGCCGAACAAATGGCTGG CCGCGATCGGCACGGTCTATGTGTCGA

[a] Mutants denoted with the same symbol (* or †) are sibling clones having identical transposon insertions

[b] Total gene sizes are: *gltJ*, 741 bp and *gltK*, 678 bp

[c] Partial sequencing results (5' to 3') obtained from arbitrary PCR and sequencing across the transposon-chromosome junction. The miniTn5 sequence (3' end) is underlined.

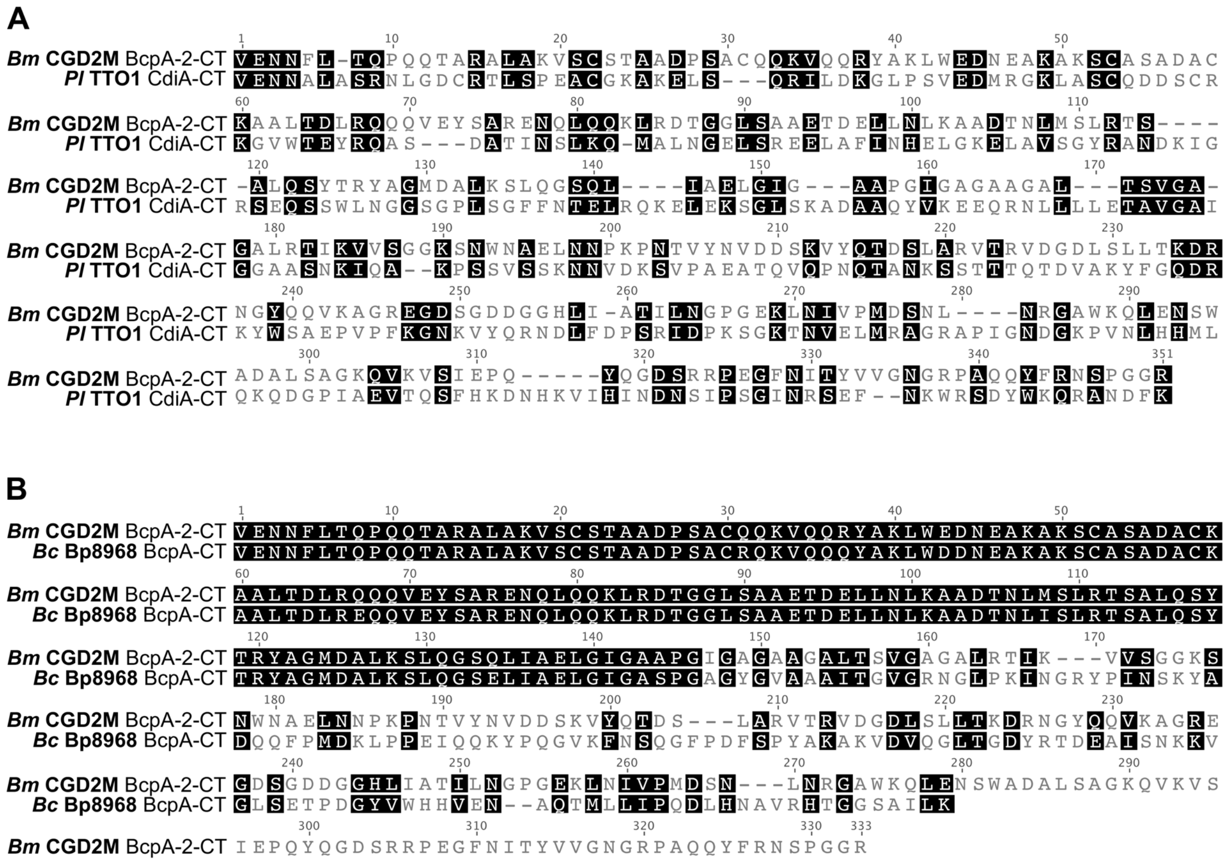

Fig. S1. Alignment of GltJK-utilizing BcpA-CT and CdiA-CT polypeptides.

A) Amino acid alignment of *Burkholderia multivorans* CGD2M BcpA-2-CT and *Photorhabdus luminescens* TTO1 CdiA-CT domains, beginning at the 'VENN' motifs.

B) Amino acid alignment of *B. multivorans* CGD2M BcpA-2-CT and *Burkholderia cenocepacia* Bp8968 BcpA-CT domains, beginning at the 'VENN' motifs.

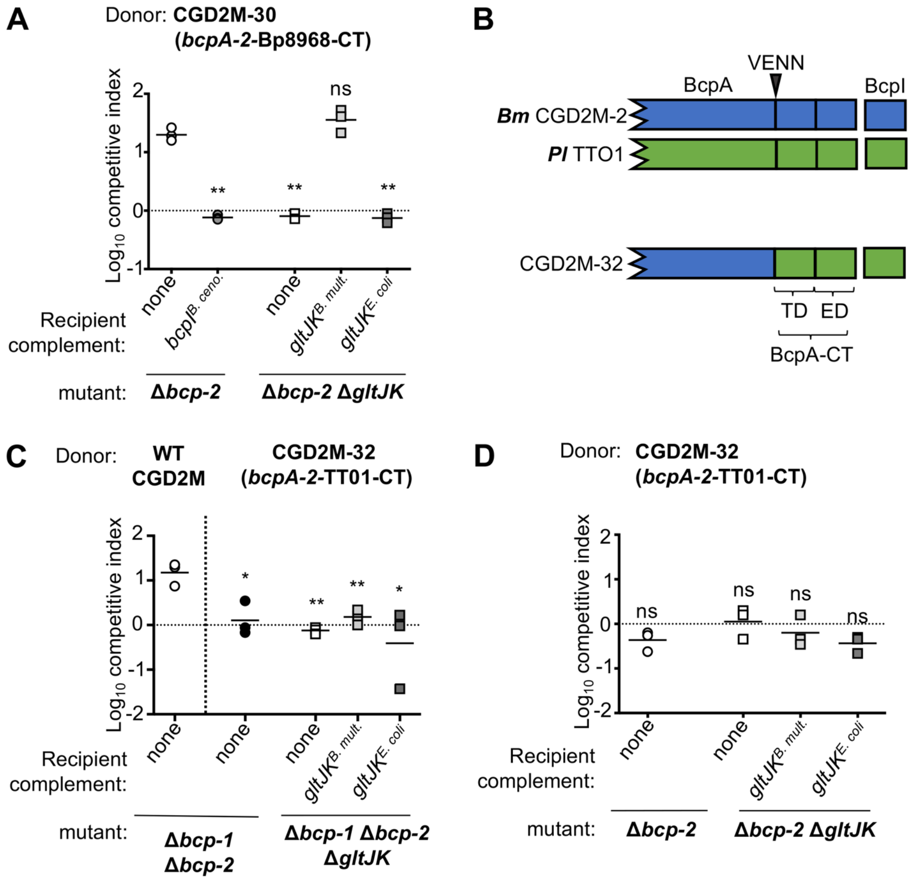

Fig. S2. Inter-bacterial competition mediated by *B. multivorans* producing chimeric BcpA-CT proteins.

A) Chimeric *B. multivorans* donor bacteria (CGD2M-30) producing chimeric BcpA-2 containing the BcpA-CT from *B. cenocepacia* Bp8968 (along with its cognate BcpI and an additional hypothetical ORF) was competed against Δ*bcp-2* recipient cells (open circles), Δ*bcp-2* recipients carrying *B. cenocepacia* Bp8968 *bcpI* at an *att*Tn7 site (gray circles), and Δ*bcp-2* Δ*gltJK* recipient cells complemented at an *att*Tn7 site with empty vector (none, white squares), or P_{S12}-driven *B. multivorans gltJK* (gray squares) or *E. coli gltJK* (dark gray squares).

B) Graphical representations of the BcpA-CT/CdiA-CT domains and BcpI/CdiI proteins from *B. multivorans* CGD2M (top), *P. luminescens* TTO1 (middle) and chimeric strain constructed (CGD2M-32, bottom). Arrow indicates the location of the VENN motif and theoretical N-terminal

end of the BcpA-CT region. Approximate locations of the putative translocation domain (TD) and effector domain (ED) of the BcpA-CT are shown.

C) Wild-type donor bacteria (left panel) or donors were competed against Δ*bcp-1* Δ*bcp-2* recipient cells. *B. multivorans* donor bacteria producing chimeric BcpA-2 containing the CdiA-CT from *P. luminescens* TTO1 along with its cognate CdiI (CGD2M-32; right panel) were competed against Δ*bcp-1* Δ*bcp-2* recipient cells (black circles) or Δ*bcp-1* Δ*bcp-2* Δ*gltJK* recipient cells carrying empty vector (none; white squares), *B. multivorans gltJK* (gray squares), or *E. coli gltJK* (dark gray squares) at an *att*Tn7 site.

D) Chimeric *B. multivorans* donor bacteria (CGD2M-32) producing BcpA-2 containing the CdiA-CT from *P. luminescens* TTO1 (along with its cognate CdiI) were competed against Δ*bcp-2* carrying empty vector (none, white circles) and Δ*bcp-2* Δ*gltJK* recipient cells carrying empty vector (none, white squares), *B. multivorans gltJK* (gray squares), or *E. coli gltJK* (dark gray squares) at an *att*Tn7 site. Competitive indices (CI) for bacteria sampled from the edge of a colony biofilm after 24 h co-culture on agar are shown. CI was calculated as (output donor CFU/recipient CFU) divided by (input donor CFU/recipient CFU). Symbols represent CI from one biological replicate and bars show the mean, n=3. Dashed line shows CI = 1 (no competition). Statistical differences were determined with two-tailed Student's t-test. *p<0.05; **p<0.01; compared to corresponding recipient cells with no *bcpI*.

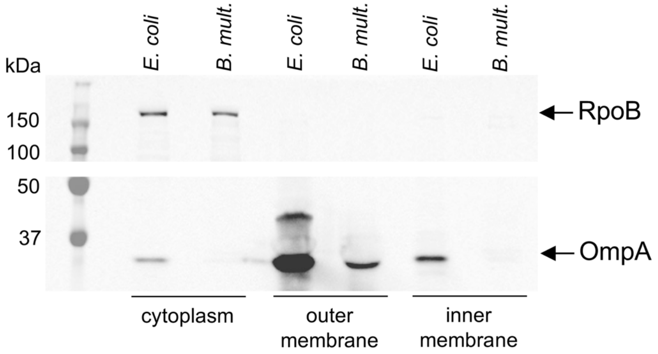

Fig. S3. Detection of putative *B. multivorans* RpoB and OmpA with anti-*E. coli* RpoB and anti- *Salmonella typhii* OmpA antibodies. Equivalent amounts of each fraction [cytoplasmic, outer membrane (Sarkosyl-soluble), and inner membrane (Sarkosyl-insoluble)] from *E. coli* or *B. multivorans* were resolved on 12% SDS PAGE and blot probed with a monoclonal antibody against *E. coli* RNA Polymerase β subunit (RpoB, top) or rabbit polyclonal against *Salmonella typhii* Outer Membrane protein A (OmpA, bottom). Expected masses for *B. multivorans* RpoB and OmpA are 153 kDa and 35kda respectively.

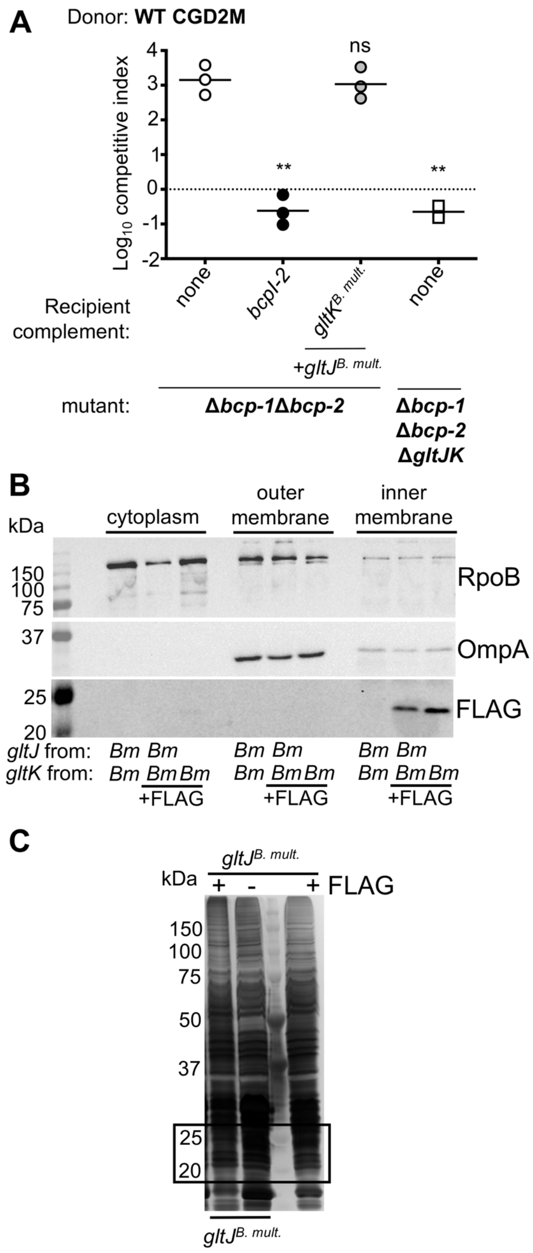

Fig. S4. Interbacterial competition with overproduction of GltJK and subcellular localization of *B. multivorans* GltK in the presence and absence of GltJ*.*

A) Replicate of experiment in Fig. 5D using different antibiotic markers to mark donor and recipient cells. Wild-type donor bacteria were competed against Δ*bcp-1* Δ*bcp-2* recipient cells (open circles), Δ*bcp-1* Δ*bcp-2* recipients carrying *bcpI-2* at an *att*Tn7 site (black circles) or P_{S12}-driven *B. multivorans gltJ* and *gltK* at each of two *att*Tn7 sites (gray circles), and Δ*bcp-1* Δ*bcp-2*

Δ*gltJK* recipient cells complemented at an *att*Tn7 site with empty vector (none, white squares). Competitive indices (CI) for bacteria sampled from the edge of a colony biofilm after 24 h co-culture on agar are shown. CI was calculated as (output donor CFU/recipient CFU) divided by (input donor CFU/recipient CFU). Symbols represent CI from one biological replicate and bars show the mean (*n*=3). Dashed line shows CI = 1 (no competition). Statistical differences were determined with two-tailed Student's t-test. ***p*<0.01, ns, not significant, compared to corresponding recipient cells with no *bcpI* unless otherwise indicated.

B) Western blots of subcellular fractions of Δ*bcp-1* Δ*bcp-2* Δ*gltJK* bacteria complemented at one of two *att*Tn7 sites with the indicated P_{S12}-driven *gltK* allele: *B. multivorans gltK*-FLAG or *B. multivorans gltK* (untagged) in the presence or absence of P_{S12}-driven *B. multivorans gltJ* (untagged) in an additional *att*Tn7 site. Equal amounts of each fraction [cytoplasmic, outer membrane (Sarkosyl-soluble), and inner membrane (Sarkosyl-insoluble)] were resolved on 12% SDS-PAGE gels and blots probed with anti- FLAG peptide (bottom), anti-*E. coli* RNA Polymerase β subunit (RpoB; top), and anti-*Salmonella typhii* Outer Membrane protein A (OmpA, middle) antibodies. Expected masses for GltK, OmpA and RpoB are ~26 kDa, ~35kDa and 150 kDa, respectively.

C) Equal amounts of the inner membrane fraction samples shown in B (derived from Δ*bcp-1* Δ*bcp-2* Δ*gltJK* mutant bacteria carrying the indicated *gltJ* and *gltK* alleles) were resolved on 12% SDS-PAGE and visualized by silver staining. Boxed region indicates region shown in in FLAG Western Blot in **B)**.

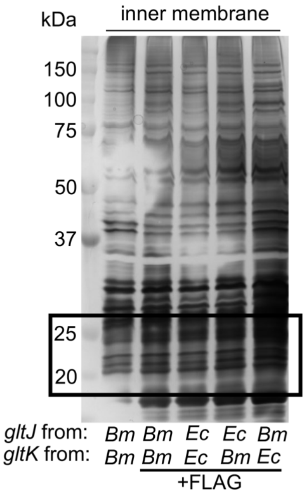

Fig. S5. Total inner membrane protein from subcellular fractionation of *B. multivorans* producing heterologous and chimeric GltK-FLAG proteins. Equal amounts of the inner membrane (Sarkosyl-soluble) fraction samples shown in Figure 7 (derived from Δ*bcp-1* Δ*bcp-2* Δ*gltJK* mutant bacteria carrying the indicated *gltJ* and *gltK* alleles) were resolved on 12% SDS-PAGE and visualized by silver staining. Boxed region indicates region shown in in FLAG Western Blot in Figure 7.

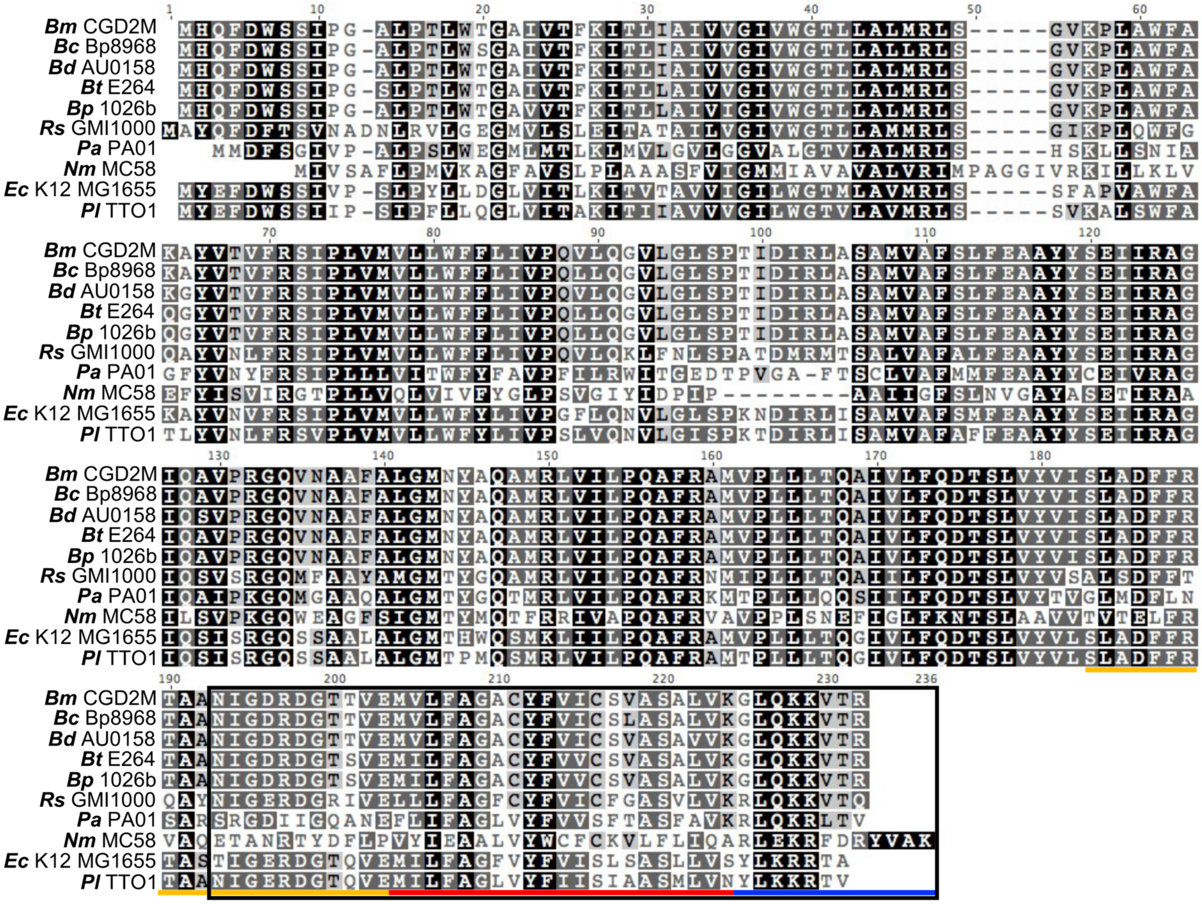

Fig. S6. Alignment of GltK proteins from selected β- and γ-proteobacterial species. Amino acid alignment of GltK from *B. multivorans* CGD2M, *B. cenocepacia* Bp8968, *Burkholderia dolosa* AU0158, *B. thailandensis* E264, *Burkholderia pseudomallei* 1026b, *Ralstonia solanacearum* GMI1000, *Pseudomonas aerugionosa* PAO1, *Neisseria meningitidis* MC58, *E. coli* MG1655, and *P. luminescens* TTO1. Boxed region denotes the C-terminal region altered in the chimeric GltK proteins (included in GltK^{Chim6}). Colored underlining indicates protein topology predicted by UniProt: yellow, predicted periplasmic region; red, predicted transmembrane helix; and blue, predicted cytoplasmic region.
